# Publisher Correction: GLP-1R–GIPR–PPARα/γ/δ quintuple agonism corrects obesity and diabetes in mice

**DOI:** 10.1038/s41586-026-10619-z

**Published:** 2026-05-18

**Authors:** Daniela Liskiewicz, Aaron Novikoff, Ahmed Khalil, Seun Akindehin, Jonathan E. Campbell, Pietra Candela, Russell L. Castelino, Callum Coupland, Maxime Culot, W. Scott Dodson, Jonathan D. Douros, Hannes Embring, Annette Feuchtinger, Brian Finan, Cristina Garcia-Caceres, Xiao-Bing Gao, Fabien Gosselet, Gerald Grandl, Robert M. Gutgesell, Daniel T. Haas, Martin Jastroch, Ezgi Karaoglu, Pamela Kakimoto, Anna Cristina Kaltenbach, Michaela Keuper, Christine M. Kusminski, Danielle C. Leander, Arkadiusz Liskiewicz, Xue Liu, Gandhari Maity-Kumar, Sara Martinez Martinez, Stephanie A. Mowery, Ruben Nogueiras, Marshall Paisley, Diego Perez-Tilve, Patricia S. S. Petersen, Paul T. Pfluger, Sneha Prakash, Sabine Steffens, Alberto Cebrian-Serrano, Monica Tost, Jordan Wean, Christian Weber, Junichi Yoshida, Zachary Gerhart-Hines, Tamas L. Horvath, Philipp E. Scherer, Randy J. Seeley, Richard D. DiMarchi, Matthias H. Tschöp, Natalie Krahmer, Patrick J. Knerr, Timo D. Müller

**Affiliations:** 1 Institute for Diabetes and Obesity, Helmholtz, Munich, Germany; 2 German Center for Diabetes Research (DZD), Munich, Germany; 3 Institute of Physiotherapy and Health Sciences, Academy of Physical Education, Katowice, Poland; 4 Department of Pharmacology and Cancer Biology, Duke University, Durham, NC USA; 5 Duke Molecular Physiology Institute, Department of Medicine, Division of Endocrinology, Duke University, Durham, NC USA; 6 UR 2465, Laboratoire de la Barrière Hémato-Encéphalique (LBHE), Université Artois, Lens, France; 7 Indiana Biosciences Research Institute, Indianapolis, IN USA; 8 Novo Nordisk Research Center Indianapolis, Indianapolis, IN USA; 9 Novo Nordisk Foundation Center for Basic Metabolic Research, Faculty of Health and Medical Sciences, University of Copenhagen, Copenhagen, Denmark; 10 Core Facility Pathology and Tissue Analytics, Helmholtz, Munich, Germany; 11 Medizinische Klinik und Poliklinik IV, Klinikum der Universität, Ludwig-Maximilians-Universität München, Munich, Germany; 12 Department of Comparative Medicine, Yale University School of Medicine, New Haven, CT USA; 13 Institute of Computational Biology, Helmholtz Munich, Munich, Germany; 14 Department of Molecular Biosciences, The Wenner-Gren Institute, Stockholm University, Stockholm, Sweden; 15 Department of Pharmacology, Experimental Therapy and Toxicology, Institute for Experimental and Clinical Pharmacology and Pharmacogenomic, Eberhard Karls University, Interfaculty Center of Pharmacogenomic and Drug Research, Tübingen, Germany; 16 Institute for Cardiovascular Prevention (IPEK), University Hospital, LMU Munich, Munich, Germany; 17 Touchstone Diabetes Center, The University of Texas Southwestern Medical Center, Dallas, TX USA; 18 Department of Physiology, Faculty of Medical Sciences in Katowice, Medical University of Silesia, Katowice, Poland; 19 CIMUS, University of Santiago de Compostela, Instituto de Investigación Sanitaria, Santiago de Compostela, Spain; 20 Department of Pharmacology and Systems Physiology, University of Cincinnati College of Medicine, Cincinnati, OH USA; 21 Research Unit Neurobiology of Diabetes, Helmholtz Munich, Neuherberg, Germany; 22 Division of NeuroBiology of Diabetes, TUM School of Medicine and Health, Technical University Munich, Munich, Germany; 23 Institute for Diabetes and Cancer (IDC), Helmholtz Diabetes Center (HDC), Helmholtz Zentrum München (Helmholtz Munich), Munich, Germany; 24 DZHK (German Centre for Cardiovascular Research) Partner Site, Munich Heart Alliance, Munich, Germany; 25 Department of Surgery, University of Michigan, Ann Arbor, MI USA; 26 Department of Biochemistry, Cardiovascular Research Institute Maastricht (CARIM), Maastricht University, Maastricht, The Netherlands; 27 Munich Cluster for Systems Neurology (SyNergy), Munich, Germany; 28 Department of Anatomy and Histology, University of Veterinary Medicine, Budapest, Hungary; 29 Department of Chemistry, Indiana University, Bloomington, IN USA; 30 Helmholtz Munich, Munich, Germany; 31 Ludwig-Maximilians-University (LMU) Munich, Munich, Germany; 32 Metabolic Cell Architecture, Department of Molecular Life Sciences, TUM School of Life Sciences, Technical University of Munich, Munich, Germany; 33 Walther-Straub Institute of Pharmacology and Toxicology, Ludwig-Maximilians-University (LMU) Munich, Munich, Germany

**Keywords:** Drug discovery, Obesity, Diabetes

Correction to: *Nature* 10.1038/s41586-026-10427-5 Published online 29 April 2026

In the version of this article initially published, there were errors in the Fig. 4d colour key labels, where the label now reading “GLP-1-GIP–Lani” alongside the red filled circle originally indicated “GLP-1”, while the label now reading “GLP-1–Lani” alongside the white open circle was originally written “GLP-1-GIP–Lani”. The figure is now amended in the HTML and PDF versions of the article.

