# Supplementary material for: GLP-1R–GIPR–PPARα/γ/δ quintuple agonism corrects obesity and diabetes in mice
Source: Nature. 2026 Apr 29;653(8115):776–85. doi: 10.1038/s41586-026-10427-5 (PMC13190304; doi:10.1038/s41586-026-10427-5)
Supplement: Supplementary file 1 — This file contains Supplementary Methods used in the manuscript and Supplementary Table 2 with all primer sequences used in the manuscript. [file 41586_2026_10427_MOESM1_ESM.docx]

**Supplementary Information 1.**

1. **Supplementary Methods**

*Generation of double incretin receptor knockout mice*

Single guide RNAs targeting exons 4 and 5 of the *Gipr* and *Glp-1r* genes were designed using the CRISPOR tool (www.crispor.tefor.net), in vitro transcribed (E3322, NEB), and purified using the MEGAclear Kit (ThermoFisher Scientific). C57BL/6n females, aged 4 weeks, were superovulated and mated with C57BL/6n males. Fertilized oocytes were collected and electroporated (NEPA21) with 200 ng/μl of sgRNAs (total concentration) and 200 ng/μl recombinant NLS-Cas9 protein (PNA Bio Inc.). Electroporated zygotes were surgically implanted into pseudopregnant CD1 recipient females. For genotyping, ear biopsies from resulting mice were lysed under standard conditions and genomic DNA was purified. The target genes were amplified using primers listed in Supplementary Table 2 and subjected to Sanger sequencing to confirm the CRISPR-induced deletions. Founders with the following deletion alleles were bred to generate double knockout mouse lines: a 5-nucleotide deletion in exon 5 of the *Gipr* gene, and deletions spanning intron 4 and exon 5 of *Glp-1r*. CRISPR-Cas9-induced deletions resulted in premature stop codons in exon 5 (*Gipr*) and exon 6 (*Glp-1r*). *Glp-1r* deletions were genotyped by PCR and resolved on agarose gel electrophoresis until double bands were obtained. To genotype the Gipr deletion, allele-specific primers targeting either the wild-type or deleted region were designed and established (*Gipr* -9nt and WT primers). All genotyping primers and single guide RNAs sequences are listed in Supplementary Table 2.

*Development of incretin-based PPAR conjugates*

Tesaglitazar (Tesa) and Lanifibranor (Lani) were purchased from MedChemExpress or Ambeed and used without further preparation. Peptide backbones were assembled via standard Fmoc/tBu solid-phase peptide synthesis using Rink amide resin, diisopropylcarbodiimide (DIC)/OxymaPure in N,N-dimethylformamide (DMF) for amino acid activation, and 20% v/v piperidine in DMF for Fmoc deprotection. To permit site-specific, on-resin conjugation of PPAR agonists **(Extended Data Figure 1a,c-e)**, the ε-amine of the C-terminal lysine residue was protected with a 4-methyltrityl group, and the α-amine of the N-terminal residue was Boc-protected. Following completion of peptide backbone assembly, the 4-methyltrityl group was removed selectively by treating the peptidyl resin with 30% v/v 1,1,1,3,3,3-hexafluoropropanol in dichloromethane. Fmoc-Glu-OtBu was then coupled and its Fmoc protection removed as described above. Tesa or Lani was then activated and coupled as above. Following completion of synthesis, compounds were cleaved from resin with 95:2.5:2.5 trifluoroacetic acid/water/triisopropylsilane, precipitated with diethyl ether, isolated by centrifugation, and purified by reversed-phase high performance liquid chromatography (RP-HPLC) with a gradient of water/acetonitrile containing 0.1% trifluoroacetic acid. Fractions containing the desired compounds in at least 90% purity as determined by analytical RP-HPLC-mass spectrometry were pooled and lyophilized to afford white powders.

Evaluation of BBB permeably in human in vitro BBB model

The in vitro BBB model was established using CD34+ endothelial cells (ECs) derived from hematopoietic stem cells, grown on matrigel-coated (BD Biosciences) insert (3401, Corning inc., NY, USA) and co-cultured with brain pericytes^57^. Hematopoietic stem cells were isolated from human umbilical cord blood collected with written and informed consent in compliance with French legislation and in line with the protocol approved by the French Ministry of Higher Education and Research (reference; CODECOH DC2011-1321). For assessment of BBB permeability, GLP-1:GIP and GLP-1:GIP:Lani were labelled with a fluorescent probe (Cyanine 5) and evaluated in comparison to ^14^C-radiolabelled sertraline and diazepam (Perkin ELMER, Boston, USA) isotopically diluted with sertraline (#S6319; Sigma Aldrich) and diazepam (#439-14-5; Sigma Aldrich). Compounds were dissolved in Ringer- Hepes (RH) Buffer (NaCl 150 mM, KCl 5.2 mM CaCl₂ 2.2 mMMgCl₂ 0.2 mM, NaHCO₃6 mM, Glucose 2.8 mM, HEPES 5 mM, water for injection, pH 7.4), to reach final concentrations of 2 μM. Sodium fluorescein (1 µM) was added as a paracellular permeability marker. To prevent non-specific binding to cell culture materials RH buffer for GLP-1 compounds was supplemented with 0.5% (w/v) human serum albumin. Reference or GLP-1 compounds were applied in the upper compartment (i.e donor) of the BBB model and incubated for respectively 1 or 3 h at 37 °C with a shaking velocity of 60 rpm (n=3 independent experiments), after which aliquots were taken from the donor and receiver compartment. The permeability to Sodium fluorescein and to the GLP-1:GIP and GLP-1:GIP:Lani was calculated based on the fluorescence quantified using the SynergyTM H1 reader (BioTek Instruments, Winooski, USA, excitation/emission wavelengths: 432/538 nm for sodium fluorescein and 650/670 nm for Cyanine 5). For sertraline and diazepam quantification was based on the ^14^C-isotope, decay per minute measured using an HIDEX 300 SL scintillation counter (Sciencetec, Villebon-sur-Yvette, France). As controls, BBB tightness (based on sodium fluorescein permeability) and compound permeability and recovery across cell-free inserts were assessed prior to the calculation of experimental permeability data.

Sodium fluorescein permeability was also used as a marker of the integrity of the BBB in the presence of either GLP-1:GIP, GLP-1:GIP:Lani or reference compounds, where an increase in the permeability of sodium fluorescein indicates a toxic effect of the studied compound on the cell layer. The raw data were computed to generate the endothelial permeability (Pe, cm/min) according to the following equations:

$\frac{1}{P_{\mathrm{se}}}=\frac{1}{P_{\mathrm{st}}}- \frac{1}{P_{\mathrm{sf}}}$ and  $P_{e}= \frac{P_{\mathrm{se}}}{S}$ Where P_se_ is the permeability surface area product across the endothelial cell layer, P_st_ is the total permeability surface area product across the endothelial cell layer and coated-filter insert, P_sf_ is the permeability surface area product across the coated-filter insert, and S is the surface area of the Transwell insert (i.e. 1.12 cm^2^).

*Indirect calorimetry and assessment of body composition*

Energy expenditure, food intake, respiratory exchange ratio (RER), and locomotor activity were assessed for 6 consecutive days, and after 24 h of acclimatization, in single-housed mice using the Promethion climate-controlled indirect calorimetric system (Sabel Systems, Las Vegas, USA). Data for energy expenditure were analyzed using ANCOVA with body weight as a covariate^58,59^**.** Fat and lean tissue mass were measured via nuclear magnetic resonance technology (EchoMRI, Houston, USA).

*Bioluminescence energy transfer (BRET) assays*

Twenty-four hours following transfection, HEK293T cells were washed with PBS and resuspended in FluoroBrite phenol red-free complete media (#A1896701, Life Technologies, Darmstadt, Germany) containing 5% FBS and 2 mM of l-glutamine (#25030081, Life Technologies, Darmstadt, Germany). 100,000 cells per well were plated into poly-d-lysine-coated (#P6403, Sigma-Aldrich, Taufkirchen, Germany) 96-well white polystyrene LumiNunc plates (#10072151, Thermo Fisher Scientific, Germering, Germany). After 24 h, the media was replaced with PBS (#10010056, Thermo Fisher Scientific, Germering, Germany) containing 10 μM of coelenterazine-h (#S2011, Promega, Walldorf, Germany) or NanoGlo (#N1110, 1:500, Promega, Walldorf, Germany). BRET measurements were taken every 30 sec for 2 min using a PHERAstar FS multi-mode microplate reader. Baseline measurements were taken after an initial 5 min of incubation with coelenterazine-h or NanoGlo-containing PBS after which cells were then treated with either vehicle (PBS) or respective ligands. The resulting ligand-specific ratiometric BRET signals were normalized to vehicle producing the ‘ligand-induced BRET ratio^60^, followed by an additional normalization step to well-specific baseline readings. Positive or negative incremental AUC (+iAUC/−iAUC) were calculated where noted. Each experiment was independently performed at least three times, each with at least three technical replicates for each group.

*In vitro quantification of PPAR-responsive genes*

Using stable hGLP-1R+ and hGIPR+ HEK293T cell lines, 4-6 distinct biological replicates of each cell line were cultured in DMEM supplemented with 10% heat-inactivated FBS, 100 IU ml−1 of penicillin and 100 μg ml−1 of streptomycin solution in separate T175 flasks (#83.3912.302, Sarstedt, Nümbrecht, Germany). Cells from each flask were seeded into 24-well plates (#10377841, Thermo Fisher Scientific, Germering, Germany) at a density of 175,000 cells/well. After 24 hrs, cells were co-transfected with 1000 ng of plasmid DNA encoding hRXRα (pcDNA3.1-hRXRα, a gift from Catharine Ross, Addgene plasmid # 135400) and one of the following: hPPARα (pcDNA3.1-hPPARα, a gift from Claes Wadelius, Addgene plasmid #169019), mPPARγ (pcDNA3.1-mPPARγ, a gift from Jaewhan Song, Addgene plasmid #78768), or hPPARδ (pAdTrack-hPPARδ, a gift from Bert Vogelstein, Addgene plasmid #16529). Twenty-four hours post-transfection, cells were then treated with respective agonists for an additional 24 hrs. After treatment, cells were flash-frozen and subsequently underwent RNA extraction, cDNA synthesis, and qPCR, as described above. Primers for qPCR are listed in **Supplementary Table 2**.

*Proteomics*

Tissues were homogenized in 2% SDC buffer (2% SDC and 100 mM Tris-HCl, pH 8.5) using a TissueLyser II (Qiagen, Hilden, Germany) with a 5-mm stainless-steel beads for 2 cycles of 2 min at 30 Hz, boiled for 10 min at 95 °C and 1,000 rpm in a thermoblock, and sonicated (Diagenode Bioruptor, 15 × 30 s). Twenty-five to fifty micrograms of proteins were reduced and alkylated with 10 mM TCEP and 40 mM chloroacetamide at 40 °C in the dark for 10 min, and digested overnight (37 °C, 1,000 rpm) with a 1:50 ratio (protein:enzyme) of trypsin (#t6567, Sigma Aldrich, Taufkirchen, Germany) and LysC (#129-02541, Wako Chemicals, Neuss, Germany). Peptides were acidified by adding 1:1 (v:v) of isopropanol and 2% TFA, and loaded onto activated triple-layer styrenedivinylbenzene reversed-phase sulfonated Stage Tips (3M Empore). Peptides were washed with ethylacetate 1% TFA, 30% methanol 1% TFA and 0.2% TFA and eluted with elution buffer (80% ACN and 5% NH4OH). Peptides were dried and dissolved in loading buffer (2% ACN and 0.1% TFA). LC–MS/MS analysis of 500 ng peptides was performed on a Orbitrap Exploris 480 (Thermo Fisher Scientific, Karlsruhe, Germany) equipped with a nano-electrospray ion source and FAIMS (CV50) coupled with an EASY-nLC 1200 HPLC (all Thermo Fisher Scientific, Karlsruhe, Germany). Peptides were separated at 60°C on 50 cm columns with an inner diameter of 75 μm packed in-house with ReproSil-Pur C18-AQ 1.9 μm resin (Dr. Maisch) over 1 h by reversed-phase chromatography using a binary buffer system consisting of buffer A (0.1 formic acid) and buffer B (80% ACN and 0.1% formic acid). Starting with 5% of buffer B, this fraction was increased stepwise to 45% over 45 min followed by a washout at 95%, at a constant flow rate of 300 nl min−1. Peptides were ionized and transferred from the LC system into to the gas phase using electrospray ionization. A DIA tandem mass spectrometry 1 h method was used^61^.

Data-independent acquisition (DIA) raw files were processed using Spectronaut software (Spectronaut v. 19.5, Biognosys) with directDIA+ and searched against the UniProt mouse databases: UP000000589_10090 and UP000000589_10090_additional, using standard parameters (trypsin cleavage with a peptide length ranging from 7 to 52 amino acids, allowing up to 2 missed cleavages). Fixed modification settings included carbamidomethylation and variable modifications were methionine oxidation and N-terminal acetylation. The analysis specified a minimum of three and a maximum of six Best N Fragment ions per peptide. For filtering and quality control, a precursor and protein q value cutoff of 1% was applied. A global normalization of data based on median quantities was implemented to correct for any MS intensity^61^. Data analysis was conducted in Perseus (v.1.6.15). The contaminants and the protein groups detected in less than 50% of the experimental groups were excluded. Label-free quantitation (LFQ) values were log2-transformed and missing values were imputed from normal distribution (width = 0.3, downshift = 1.8). Significantly changed proteins between the conditions were determined by ANOVA (FDR 0.05). Four samples were excluded from the analysis (one per tissue: Quad, brain stem, eWAT, and hypothalamus) as they did not pass quality control. For Fisher’s exact tests, annotations were extracted from UniProtKB, Gene Ontology (GO), and the Kyoto Encyclopedia of Genes and Genomes (KEGG) using Perseus (enrichment factor > 1.5 or 1.3) or Gprofiler (term coverage >15%)^62^. Complete enrichment analyses are provided in the data source file and representative categories are visualized.

*Bulk RNA sequencing*

Bulk RNA sequencing was performed from samples corresponding to **Figure 2a-f.** Samples were obtained 24hr after the last drug injection to avoid assessment of acute drug effects. Total RNA was extracted using the RNeasy Kit (QIAGEN, Hilden, Germany) according to manufacturer's instructions. RNA integrity number (RIN) was measured using the Agilent 5300 Fragment Analyzer System. Samples with a RIN value > 7 were selected for mRNA sequencing (poly-A selected). The libraries were prepared using the Illumina stranded mRNA prep ligation kit with an input of 1000ng of total RNA, following the kit's instructions. After a final quality control, the libraries were sequenced in a paired-end mode (2x100 bases) in the NovaseqX+ sequencer (Illumina) with a depth of ≥ 50 Million reads per sample. FASTQ files were generated from base calls using the bcl2fastq tool from Illumina bcl2fastq Conversion Software (bcl2fastq version: 2.17.1.14). Library preparation, sequencing, and demultiplexing was performed at the Helmholtz Zentrum München (HMGU) by the Genomics Core Facility.

Quality control for the raw transcriptomics sequencing reads was done using FastQC^63^, then transcripts were aligned and quantified using the GRCm38 reference genome (GenBank accession GCA_000001635.20) using STAR^64^ with default parameters. Transcript counts were imported into python using scanpy^65^ and decoupler^66^, where the python implementation of DESeq2^67^, pyDESeq^68^, was used to normalize gene expression with the median ratio method, principal components were calculated using normalized counts. Differentially expressed genes (DEGs) were identified using standard parameters and a two-group comparison Wald test. Gene expression z-scores of DEGs were plotted using Matplotlib (doi.org/10.5281/zenodo.592536), ggplot2^69^ and seaborn^70^. Functional enrichment scores of hallmark gene sets from the molecular signatures database^71^ were inferred using the Over Representation Analysis (ORA) method implemented in decoupler^66^.

*Immunofluorescence*

For the assessment of the number of c-Fos positive cells, mice were treated s.c. with a single injection of either GLP-1:GIP, GLP-1:GIP:Lani or Lani (50 nmol/kg). 90 min after drug administration, mice were euthanised by CO_2_ and perfused with ice-cold TBS followed fixation using Roti®-Fix (Carl Roth GmbH, Karlsruhe, Germany). Twenty-four hours post-fixation, brains were coronally cryosectioned into 35 μm thick slices and immunolabeled with a monoclonal rabbit anti-c-Fos antibody (#MA5-15055, 1:400, Invitrogen, Karlsruhe, Germany) and the anti-rabbit Alexa546 secondary antibody (#A10040, 1:2,000, Invitrogen, Karlsruhe, Germany). According to the Allen Mouse Brain Atlas, the dorsal vagal complex (DVC), containing the area postrema (AP) and the nucleus tractus solitarius (NTS), was captured using a ×20 objective in z-stack mode with a Leica SP8 confocal microscope and imaged using LAS X (v.3.5.7.23225, Leica Microsystems, Wetzlar, Germany). In each region, the number of cFos-positive cells was automatically counted by using QuPath v.0.4.4 software^72^. For c-Fos quantification in hypothalamus, whole brain slices were scanned in z-stack mode with AxioScan 7 digital slide scanner (ZEN Blue v.3.5, ×20 objective, Zeiss, Oberkochen, Germany). The Allen Brain Atlas was imputed and aligned to the whole slide images using Fiji with the ABBA plugin and the number of c-Fos-positive cells in dorsomedial hypothalamus (DMH), ventromedial hypothalamus (VMH) and arcuate nucleus (ARC) was measured by using QuPath.

*Hyperinsulinemic euglycemic clamps*

Wildtype male DIO C57BL/6J mice were purchased from the Jackson Laboratories (Bar Harbor, ME, USA, # 380050). At the age of 26-28 weeks, mice underwent dual cannulation surgery as described previously^73^**.**Animals received Carprofen (0.5 mg/kg) and Gentamicin (0.8 mg/kg) subcutaneously prior to the start of surgery. Post-surgical recovery lasted eight days, body weight was checked daily, and cannula patency was checked with 200 U/mL Sodium Heparin solution daily for the first three days and then every 48 hours until time of clamps. On day eight or nine post-recovery, mice were weighed and grouped. Animals who lost >20% pre-surgical body weight were removed from the study. The day after the final injection of the 5-day treatment, animals were transferred from their home cages to the clamp room and fasted with ad lib access to water. After 3 hours of fasting, animals were connected to a sterile saline primed tether and swivel (Instech Laboratories, PA, USA) and allowed 20 minutes to acclimate to the connection. Baseline glycemia and whole blood was collected through the arterial line, and a bolus infusion of 6,6-D2-Glucose (Cambridge Laboratories, OH, USA) was infused over 5 minutes at 9.82 mg/kg/min and then changed to a continuous infusion of 0.492 mg/kg/min for a total of 120 minutes. Samples were collected at 90, 100, 110, and 120 minutes to measure basal endogenous glucose production. After a total of 5 hours of fasting, animals were connected to a cocktail mixture of D50 and 6,6-D2-Glucose, insulin (4 mU/kg/min), and saline washed donor red blood mixed in 10U/mL Heparin^73^. The starting glucose infusion rate (GIR) was 20-25 mg/kg/min for each animal. Glycemia was measured every 10 minutes, and GIR was adjusted to maintain euglycemia at a range of 140-160 mg/dL. Whole blood for steady-state endogenous glucose production was collected at 90, 100, 110, and 120 minutes.

*LCMS for 6,6-D2-Glucose labeling in Plasma*

5 uL of plasma was added to an Eppendorf tube and spiked with 5 uL of 5mM 13C6 Glucose as an internal standard. Methanol, water and chloroform (400 ul each) was added sequentially to the tube with vortexing after each addition. The sample was centrifuged at 13,000 RPM for 15 minutes, and 700 uL of the supernatant was transferred to a new tube. The supernatant was dried under N2 gas, and 100 uL H_2_O was added to resuspend the pellet after which 50 uL of the sample to LC-MS vials for analysis.

LC-Q-Exactive+-Orbitrap-MS was used for glucose labeling assay. The Vanquish Binary Pump was used to deliver mobile phase (98% H2O and 2% methanol containing 0.01% formic acid) at a flow rate of 0.5 ml/min in an isocratic elution mode. The column was a Microsorb-MV C18 column (100 × 4.6 mm, 3 µm) with a C18 guard column and was kept at 40 °C in a column oven compartment. The autosampler was maintained at 5 °C and the injection volume was 1 µl. The total running time was 10 minutes. The parameters for Q-Exactive+-MS equipped with a HESI probe were set at following: heat temperature: 425 °C, sheath gas: 30, auxiliary gas: 13, sweep gas: 3, spray voltage: 3.5 kV in positive mode, capillary temperature: 320 °C, and S-lens was 45. A full m/z scan range was from 60 to 900. The resolution was set at 70,000 at m/z of 200. The maximum injection time (max IT) was 200 ms. Automated gain control (AGC) was targeted at 3 × 10^6^ ions.

*Calculations*

Glucose rate of appearance (R_a_) was calculated using a modified version of Steele’s non-steady state equation published by Cowen and Hetenyi^74^. In brief, pool fraction was constant at 0.65 and the volume distribution was set at 2 dL/kg. *E* is the enrichment fraction at each timepoint, and *G* is the glucose value (mg/dL) at each timepoint. *TIR* is the tracer infusion rate at each time point. Endogenous glucose production (EGP) was calculated by subtracting the glucose infusion rate (mg^-1^kg^-1^min^-1^) from the rate of appearance.

$$R_{a} \left( {mg}^{-1} {kg}^{-1}{min}^{-1} \right)=\frac{TIR \left( {mg}^{-1} {kg}^{-1}{min}^{-1} \right)}{Average\left( E_{1}:E_{2} \right)}-pV*\frac{Average\left( G_{1}:G_{2} \right)}{Average\left( E_{1}:E_{2} \right)}*\Delta E\Delta t$$

*Tissue-selective glucose uptake*

Mice were fasted for 6 hours, then anaesthetized with 75 mg/kg pentobarbital and placed on heating pads (37°C). 0.33 μCi/g BW 3H-labeled 2-deoxyglucose (3H-2DG) (#ART0103, Hartmann Analytics) in 20% glucose solution was injected retro-orbitally to a final dose of 1 g/kg BW glucose. To determine plasma decay of 3H, tail vein blood samples (10 μL) were collected at times 0, 5, 10, 15, 20 and 25 minutes post injection. Additionally, blood glucose levels were measured at each timepoint using a CONTOUR®NEXT EZ meter and glucose indicator strips (#84167836, Bayer). Blood samples were immediately mixed with 45 μL BaOH (#B4059, Sigma), and 45 μL of ZnSO4 (#Z2876, Sigma) was mixed in to precipitate protein, before being placed on ice. Samples were centrifuged (5 min, 14000 g, 4°C) and 50 μL of the supernatant was transferred into 3 mL of Ultima Gold (#6013329, PerkinElmer) and counted on HIDEX 600SL (HIDEX) for 10 min per sample. Mice were euthanized by cervical dislocation 25 min post injection and tissues were snap frozen. 10-100 mg tissue was weighed out and homogenized in 800 μL cold Pierce RIPA buffer (#89900, Thermo Scientific). Samples were centrifuged (20 min, 14000 g, 4°C) and 150 μL of the supernatant was deproteinized with 600 μL 4.5% perchloric acid (PCA, diluted from Sigma, #311421). Another 150 μL was deproteinized with 300 μL BaOH and 300 μL ZnSO4 to precipitate 2-deoxyglucose-6-phosphate. Samples were vortexed and centrifuged (5 min, 14000 g), and 500 μL of the supernatant was transferred into 3 mL of Ultima Gold and counted on HIDEX 600SL for 10 min per sample. Glucose uptake was calculated based on previously established methods^75^. Background blood 2DG count (baseline) was subtracted from each blood sample count and subsequently recalibrated to account for the blood dilution factor. AUC was calculated for both the glucose measurements (mg glucose) and the 2DG counts (timepoints: 0, 5, 10, 15, 20, 25 min), and a conversion factor was calculated (AUC: DPM/mg glucose). BaOH/ZnSO4-precipitation supernatant counts were subtracted from the Perchloric extract counts to obtain the tissue-trapped glucose and the result was recalibrated to account for the tissue dilution factor and normalized to tissue weight and the conversion factor. Glucose uptake is presented as ng/mg/25 min.

*Cardiovascular drug effects*

Cardiac function and blood pressure were assessed in DIO C57BL6/J mice treated for 14 days with either vehicle or 10 nmol/kg of either GLP-1:GIP or GLP-1:GIP. Cardiac measures were assessed one day after the last injection. Anesthesia was induced with 4% isoflurane (delivered in oxygen at a flow rate of 1 L/min) and maintained at 1.5% during echocardiographic imaging. Using the Vevo 3100 High-Resolution Micro-Ultrasound Imaging System (FUJIFILM VisualSonics, Toronto, Canada) equipped with the MX550D transducer (25-55 MHz), B-mode images of the left ventricle (LV) in parasternal long axis (PSLAX) view were acquired. Ejection fraction (EF), fractional shortening (FS), cardiac output (CO), stroke volume (SV) and heart rate (HR) were quantified with the VevoLab Version 5.10.0 (FUJIFILM VisualSonics, Toronto, Canada) in AM-mode via LV trace. Isoflurane was reduced to 0.5% for blood pressure measurements using the CODA® Monitor tail-cuff system (AD Instruments, Oxford, United Kingdom), and the mean of four to five measurements was calculated with LabChart Version 8.1.24 (AD Instruments, Oxford, United Kingdom).

*Assessment of POMC neuronal activity*

For assessment of POMC neuronal activity, POMC-Cre mice (strain 005965, Jackson Laboratory; aged 10-34 wks) were maintained on a C57BL/6 genetic background. All experimental procedures were approved by the Institutional Animal Care and Use Committee (IACUC) of Yale University. The animals were housed in temperature- (20–23 °C) and humidity- (30-70%) controlled rooms, on a 12/12-hour light/dark cycle, with lights on from 07:00 to 19:00. Prior to the behavioral and fiber photometry experiments, the mice were moved to a reversed light cycle room, with lights on from 21:00 to 09:00, at least one week before the start of the experiments. Mice were provided with food and water ad libitum, except on specific days when behavioral experiments were conducted. All stereotaxic surgeries were performed under isoflurane anesthesia (4% for initial sedation, maintained at 1-2%) with regular monitoring to ensure a stable respiratory rate and absence of tail or toe pinch response. Mice were positioned in a stereotactic apparatus, and a heating pad was used to prevent hypothermia. Regional anesthesia was administered with an injection of 80 µL/10 g body weight of 0.5% Bupivacaine (Hospra), followed by 50 µL of Ethiqa XR (Fidelis Animal Health) for analgesia. After shaving the skull hair, a midline incision was made, and the skull surface was cleaned of all connective tissue. Craniotomies were performed using a drill over the designated injection sites and/or optic fiber placement locations, guided by coordinates from the Mouse Brain Atlas. A virus expressing calcium indicators (AAV9-syn-FLEX-jGCaMP8m-WPRE: 162378-AAV9, Addgene) was injected into arcuate nucleus (a single hemisphere for fiber photometry only or both hemispheres for optogenetics) at the following coordinates relative to the bregma: AP, -1.58 mm; ML, ±0.1 mm; DV, 5.7 mm. The injections were conducted using a nano injector (Nanoject III, Drummond) equipped with glass pipettes. After injection, the glass pipette was left in place for at least 5 minutes before retraction to allow for proper diffusion. A total of 900 nL of the virus solution was injected at a rate of 1 nL per second. For fiber photometry recordings, optic fibers (400 µm in diameter, 0.48 NA, Neurophotometrics) were implanted at the coordinates AP, -1.58 mm; ML, ±0.25 mm; DV, 5.65 mm, positioning the fiber tip 50 µm above the virus injection site. The fibers were secured to the skull using a stereotaxic cannula holder (XCL, Thorlabs), along with Optibond (Kerr Dental) and Charisma (Heraeus Kulzer) dental materials. For head-fixation, a metal bracket was implanted on the skull's surface using Charisma, and a final layer of dental cement was applied to secure the fibers and brackets. Following surgery, mice were administered 100 µL/10 g body weight of Meloxidyl (Ceva Animal Health) as an antibiotic. Additional doses of Metacam were given 24 and 48 hours post-surgery. Mice were allowed to recover for at least 2 weeks before starting the experiments to ensure full recovery and adequate viral expression. Calcium indicator (i.e., GCaMP8m) signals from POMC neurons were measured using a commercial fiber photometry system, the LUX RZ10X processor (Tucker-Davis Technologies). Recording and data acquisition were controlled by Synapse software (Tucker-Davis Technologies, v.102). To correct for motion artifacts in the recorded calcium indicator signals, two excitation wavelengths were utilized with 30 µW power intensity. The 465 nm LED, modulated sinusoidally at 534 Hz, excited calcium-dependent fluorescence (calcium indicator signal). Additionally, the 405 nm LED, the isosbestic wavelength for GCaMP, was modulated sinusoidally at 211 Hz to acquire calcium-independent fluorescence, serving as a control signal. Both wavelengths were combined through an optical fiber patch (Doric Systems) using a minicube (FMC4, Doric Systems) and focused on a femtowatt photoreceiver. The recorded signal was corrected using the control signal and synchronized with the behavioral experiment data. Data were acquired at a rate of 1 kHz. One day prior to recording, food pellets were removed from the home cages and mice were maintained in a fasted state. For pharmacological testing under head fixation with fiber photometry, mice were habituated to the head-fixed apparatus during the first 15 minutes of recording to minimize the initial reduction in background signal and prevent baseline drift. Following habituation, mice received a subcutaneous injection of 50 µL solution per body weight of either PBS, the GLP:GIP backbone, or the GLP:GIP:Lani compound, and fiber photometry signals were continuously recorded. Each mouse first underwent the PBS session, followed by the GLP:GIP backbone or GLP:GIP:Lani compound session after a 1-day interval. The remaining compound session was conducted after an additional 7-day washout period. Calcium indicator (i.e., GCaMP8m) signals from POMC neurons were measured using a commercial fiber photometry system, the LUX RZ10X processor (Tucker-Davis Technologies). Recording and data acquisition were controlled by Synapse software (Tucker-Davis Technologies). To correct for motion artifacts in the recorded calcium indicator signals, two excitation wavelengths were utilized with 30 µW power intensity. The 465 nm LED, modulated sinusoidally at 534 Hz, excited calcium-dependent fluorescence (calcium indicator signal). Additionally, the 405 nm LED, the isosbestic wavelength for GCaMP, was modulated sinusoidally at 211 Hz to acquire calcium-independent fluorescence, serving as a control signal. Both wavelengths were combined through an optical fiber patch (Doric Systems) using a minicube (FMC4, Doric Systems) and focused on a femtowatt photoreceiver. The recorded signal was corrected using the control signal and synchronized with the behavioral experiment data. Data analysis for fiber photometry and behavioral experiments was conducted using customized Python programs. The recorded calcium indicator signal (coming from 465 nm LED excitation) was corrected using the control signal (coming from 405 nm LED excitation) by a modified protocol described previously^76^. To remove the slope and low-frequency fluctuations in signals, fit curves were calculated and subtracted from both calcium indicator and control signals separately. The signals were standardized using the mean value and standard deviation (z-scoring: z-scored signal = ( signal - average ) / SD; zSig465 and zSig405). Using non-negative robust linear regression, fit standardized signals were fit to the regression function:

zSig465 = a * zSig405 + b. New values of zSig405 fitted to zSig465 (fitSig405) were found using the parameters a and b of the linear regression (fitSig405 = a * zSig405 + b). The normalized dF/F (z-dF/F) was calculated (z-dF/F = zSig465 - fitSig405). The normalized dF/F signal was further standardized (z-score) using the baseline period from 0–120 s after drug administration to improve visualization of signal dynamics. Fiber photometry signal magnitude was quantified as the median intensity during 200–400 s post-administration.

*Electophysiology in POMC-GFP mice*

Coronal hypothalamic slices, 300 µm thick, were cut from mice expressing GFP exclusively in POMC neurons under the control of the POMC promoter as reported previously^77^. Briefly, mice were deeply anesthetized with isoflurane and decapitated. Brains were rapidly removed and immersed in an oxygenated bath solution at 4^o^C containing (in mM): sucrose 220, KCl 2.5, CaCl_2_ 1, MgCl_2_ 6, NaH_2_PO_4_ 1.25, NaHCO_3_ 26, and glucose 10, pH 7.3 with NaOH. After preparation, slices were maintained in a holding chamber with artificial cerebrospinal fluid (ACSF) (bubbled with 5% CO_2_ and 95% O_2_) containing (in mM): NaCl 124, KCl 3, CaCl_2_ 2, MgCl_2_ 2, NaH_2_PO_4_ 1.23, NaHCO_3_ 26, glucose 10, pH 7.4 with NaOH, and were transferred to a recording chamber constantly perfused with a modified ACSF solution (with 2.5 mM glucose) (33 ^o^C) at 2 ml/min after at least a 1 hr recovery. Whole-cell current clamp was performed to monitor spontaneous action potentials (APs) in POMC-GFP neurons in the ARC with a Multiclamp 700A amplifier (Molecular Devices, CA) as described previously^77^. The patch pipettes (4-6 MΩ) were made of borosilicate glass (World Precision Instruments) with a Sutter pipette puller (P-97) and filled with a pipette solution containing (in mM): K-gluconate 135, MgCl_2_ 2, HEPES 10, EGTA 1.1, Mg-ATP 2, Na_2_-phosphocreatine 10, and Na_2_-GTP 0.3, pH 7.3 with KOH. After a giga-ohm (GΩ) seal and whole-cell access were achieved, the series resistance (between 10 and 20 MΩ) was partially compensated by the amplifier. After a stable recording of APs was achieved for at least 5 minutes, the GLP:GIP backbone (2 nM) or the GLP:GIP:Lani compound (2 nM) was applied to recorded neurons through bath solution for 5-10 minutes and then washed out with control ACSF. In all experiments, only recordings with stable series resistance were accepted (the change in series resistance was less than 20%). All data were sampled at 10 kHz and filtered at 3 kHz with an Apple Macintosh computer using AxoGraph X (AxoGraph, Inc., v. 1.7.3). AP frequency was detected and analyzed with AxoGraph X and plotted with Igor Pro software (WaveMetrics, Lake Oswego, OR) and Prism (v. 10.0.3.

*Isolation and differentiation of stromal vascular fraction cells and Oil Red O staining*

Isolation of the stromal vascular (SV) fraction from the inguinal white adipose tissue (iWAT) pad of WT mice was performed as follows: iWAT fat pads were minced and then digested for 30 min at 37°C (DMEM/F12 plus glutamax, 0.1% (w/v) collagenase Type IV, 1% BSA). The digested tissue was filtered through a 100 μm filter and rinsed with PBS. Thereafter, cells were centrifuged at 500 g for 5 min. The pellet was washed once in PBS and centrifuged again at 500 g for 5 min. Cells were re-suspended in growth media (DMEM/F12 plus glutamax, 10% Fetal bovine serum, 1% penicillin/streptomycin) plated and cultured. The next day, cells were washed twice with PBS to remove floating cells. Cells were later trypsinized and split into 48-well plates. Upon reaching confluence, Induction was induced using 850nM Insulin (Sigma-Aldrich, Missouri, USA), 5µM Dexamethasone (Sigma-Aldrich) and 0.5mM 3-Isobutyl-1-methylxanthin (Sigma-Aldrich) along with the tested drugs in growth medium. On days 2,4 and 6 of differentiation, medium was changed with growth medium containing 850nM Insulin along with the tested drugs. On day 8 of differentiation, Cells were washed with PBS, fixed with 4% paraformaldehyde for 1 hour and washed twice with water followed by a 5 min incubation with 60% isopropanol. Cells were left to dry completely and then Oil Red O (Thermo Fisher Scientific) was added for 10 min. Cells were washed with water, imaged using Invitrogen™ EVOS™ XL Core Imaging System (Thermo Fisher Scientific) and analysed using ImageJ (v. 1.54). Absorbance was measured using PHERAstar (BMG Labtech, Ortenberg, Germany).

*Extracellular Flux Measurements (XF96 Seahorse)*

Immortalized mouse brown preadipocytes^78^ were seeded (9k/well) onto Seahorse XFe96-well plates Seahorse Bioscience, Agilent Technologies). After 24h, adipogenic in vitro differentiation was induced as described previously^78^. On day 5 or 6 of adipogenic differentiation, the cells were incubated with growth medium containing 100 nM of compound or its vehicle (PBS) as control. After 24h, the cells were washed and incubated in an air incubator at 37 °C with XF assay medium (XF DMEM medium (103575-100, Agilent), supplemented with 25 mM glucose (G6152, Sigma) and 2 mM glutamine (25030024, Gibco, ThermoFisher), and 0.4% fatty-acid free BSA (A3803, Sigma), and the pH adjusted to 7.5. The adipocytes were incubated for 60 min before transferring the XF96 plate to a temperature-controlled Seahorse extracellular ﬂux analyzer (Agilent Technologies) at 37°C, incubated for temperature equilibration before starting with assay cycles consisting of 2-min mix and 2-min measure periods. After four basal assay cycles, the wells treated with 100 nM compounds or vehicle received an injection of oligomycin to inhibit the ATP synthase (4 µg/ml, O4876, Sigma), allowing to determine ATP-linked respiration using the lowest value of four cycles. The uncoupler 2,4-Dinitrophenol (2,4-DNP; 100 µM, 34334, Sigma) was injected to stimulate maximal substrate oxidation capacity, defined as the highest respiration value. Next, a mixture of rotenone (R, 4 µM, R8875 Sigma) and antimycin A (AA, 2 µM, A8674, Sigma) was added, followed by three assay cycles to determine non-mitochondrial OCR (lowest value). Finally, 2-deoxy-glucose (2-DG, 100 mM, D8375, Sigma) was added to inhibit glycolytic extracellular acidiﬁcation rates (ECARs). For the assessment of acute effects, compound (100 nM) or vehicle was injected after four basal assay cycles, followed by oligomycin, DNP and a mixture of 2-DG, rotenone and antimycin A. The ECAR values were transformed to PPR values. For ‘glycolysis’, the mean of the four basal cyles was determined. For ‘induced glycolysis’, the highest PPR value after oligomycin injection was determined. The lowest 2-DG value was subtracted to correct for non-glycolytic PPR. ‘Glycolytic PPR’ is the lowest PPR value after R/AA injection, corrected for the lowest PPR value after 2-DG injection. ATP production from glycolysis and oxphos was calculated as described previously^791^. After the measurements, the cells were lysed and total dsDNA amount per well was determined using Quant-iT PicoGreen dsDNA Assay Kit (P7589, Invitrogen, ThermoFisher). All OCR and PPR values were normalized to dsDNA content and for illustration in the graphs, OCR and PPR data were adjusted to 100 ng dsDNA.

1. **Supplementary Table 2**

Used Primer sequences

| **Target** | **Primer** |
| --- | --- |
| Gipr gRNA1 | 5´-GGCTTTGTCTTCCGCCAGTG- 3´ |
| Gipr gRNA2 | 5´-GGTCTCTCCAAGATCCCCAC- 3´ |
| Gipr gRNA3: | 5´-CTGCAGATCATGTATACCGT- 3´ |
| Gipr gRNA4 | 5´-CCTGCAGATCATGTATACCG- 3´ |
| Glp1r gRNA1 | 5´-TAGACTCTTCACACTCCGAC- 3´ |
| Glp1r gRNA2: | 5´-CTGTGCAGAACCGGTACACA- 3´ |
| Glp1r gRNA3: | 5´-CCACTGTGTAGATAATGTAC- 3´ |
| Glp1r gRNA4: | 5´-GATGGCTGAAGCGATGACCA- 3´ |
| Glp1r F: | 5´-TGCGATTCCTGTTACTAACTCA- 3´ |
| Glp1r R: | 5´-AGTGAGAAGGACCCTCTGGTT- 3´ |
| Gipr F: | 5´-AGCTGATCTCGGGTGAGGAT- 3´ |
| Gipr R: | 5´-TGTGGCGATCAGAGGTCAAC- 3´ |
| Gipr -9nt F: | 5´-TCGTCAGGGACAGGGAGTAG- 3´ |
| Gipr -9nt R: | 5´-CAGTGATGGAGTGATCTTGGAG-3' |
| Gipr WT R: | 5´-CAAGATCCCCACTGGCCATC-3' |
| Hprt F: | 5'-AAGCTTGCTGGTGAAAAGGA-3' |
| Hprt R: | 5'-TTGCGCTCATCTTAGGCTTT-3' |
| GLP-1R F: | 5'-AGCACTGTCCGTCTTCATCA-3' |
| GLP-1R R: | 5'-AGAAGGCCAGCAGTGTGTAT-3' |
| Gipr F: | 5'-GTGTCCACGAGGTGGTGTTT-3' |
| Gipr R: | 5'-CCGACTGCACCTCTTTGTTG-3' |
| PDK4 F: | 5'-AGAGGCCACTGTCGTCTTGG-3' |
| PDK4 R: | 5'-AACCAAAACCAGCCAAAGGGG-3' |
| PGC1𝛼 F: | 5'-AGCCGTGACCACTGACAACGAG-3' |
| PGC1𝛼 R: | 5'-GCTGCATGGTTCTGAGTGCTAAG-3' |
| UCP1 F: | 5'-ACTGCCACACCTCCAGTCATT-3' |
| UCP1 R: | 5'-CTTTGCCTCACTCAGGATTGG-3' |
| PRDM16 F: | 5'-CCGCTGTGATGAGTGTGATG-3' |
| PRDM16 R: | 5'-GGACGATCATGTGTTGCTCC-3' |
| Cidea1 F: | 5'-AATGGACACCGGGTAGTAAGT-3' |
| Cidea1 R: | 5'-CAGCCTGTATAGGTCGAAGGT-3' |
| PC F: | 5'-GATGACCTCACAGCCAAGCA-3' |
| PC R: | 5'-GGGTACCTCTGTGTCCAAAGGA-3' |
| PEPCK F: | 5'-CTGCATAACGGTCTGGACTTC-3' |
| PEPCK R: | 5'-CAGCAAACTCCCGTACTCC-3' |
| GIPR F: | 5'-GTGTCCACGAGGTGGTGTTT-3' |
| GIPR R: | 5'-CCGACTGCACCTCTTTGTTG-3' |

**References**

57 Cecchelli, R. *et al.* A stable and reproducible human blood-brain barrier model derived from hematopoietic stem cells. *PLoS One* **9**, e99733, doi:10.1371/journal.pone.0099733 (2014).

58 Muller, T. D., Klingenspor, M. & Tschop, M. H. Revisiting energy expenditure: how to correct mouse metabolic rate for body mass. *Nat Metab* **3**, 1134-1136, doi:10.1038/s42255-021-00451-2 (2021).

59 Tschop, M. H. *et al.* A guide to analysis of mouse energy metabolism. *Nat Methods* **9**, 57-63, doi:10.1038/nmeth.1806 (2011).

60 Porrello, E. R. *et al.* Heteromerization of angiotensin receptors changes trafficking and arrestin recruitment profiles. *Cell Signal* **23**, 1767-1776, doi:10.1016/j.cellsig.2011.06.011 (2011).

61 Klingelhuber, F. *et al.* A spatiotemporal proteomic map of human adipogenesis. *Nat Metab* **6**, 861-879, doi:10.1038/s42255-024-01025-8 (2024).

62 Raudvere, U. *et al.* g:Profiler: a web server for functional enrichment analysis and conversions of gene lists (2019 update). *Nucleic Acids Res* **47**, W191-W198, doi:10.1093/nar/gkz369 (2019).

63 Andrews, S. A quality control tool for high throughput sequence data. [*https://www.bioinformatics.babraham.ac.uk/projects/fastqc/*](https://www.bioinformatics.babraham.ac.uk/projects/fastqc/) (2023).

64 Dobin, A. *et al.* STAR: ultrafast universal RNA-seq aligner. *Bioinformatics* **29**, 15-21, doi:10.1093/bioinformatics/bts635 (2013).

65 Wolf, F. A., Angerer, P. & Theis, F. J. SCANPY: large-scale single-cell gene expression data analysis. *Genome Biol* **19**, 15, doi:10.1186/s13059-017-1382-0 (2018).

66 Badia, I. M. P. *et al.* decoupleR: ensemble of computational methods to infer biological activities from omics data. *Bioinform Adv* **2**, vbac016, doi:10.1093/bioadv/vbac016 (2022).

67 Love, M. I., Huber, W. & Anders, S. Moderated estimation of fold change and dispersion for RNA-seq data with DESeq2. *Genome Biol* **15**, 550, doi:10.1186/s13059-014-0550-8 (2014).

68 Muzellec, B., Telenczuk, M., Cabeli, V. & Andreux, M. PyDESeq2: a python package for bulk RNA-seq differential expression analysis. *Bioinformatics* **39**, doi:10.1093/bioinformatics/btad547 (2023).

69 Wickham, H. ggplot2: Elegant Graphics for Data Analysis. *Springer-Verlag New York* (2016).

70 Waskom, M. L. Seaborn: statistical data visualization. *Journal of Open Source Software* **6**, 3021, doi:doi.org/10.21105/joss.03021 (2021).

71 Liberzon, A. *et al.* The Molecular Signatures Database (MSigDB) hallmark gene set collection. *Cell Syst* **1**, 417-425, doi:10.1016/j.cels.2015.12.004 (2015).72

72 Bankhead, P. *et al.* QuPath: Open source software for digital pathology image analysis. *Sci Rep* **7**, 16878, doi:10.1038/s41598-017-17204-5 (2017).

73 Ayala, J. E., Bracy, D. P., McGuinness, O. P. & Wasserman, D. H. Considerations in the design of hyperinsulinemic-euglycemic clamps in the conscious mouse. *Diabetes* **55**, 390-397, doi:10.2337/diabetes.55.02.06.db05-0686 (2006).

74 Cowan, J. S. & Hetenyi, G., Jr. Glucoregulatory responses in normal and diabetic dogs recorded by a new tracer method. *Metabolism* **20**, 360-372, doi:10.1016/0026-0495(71)90098-9 (1971).

75 Ferre, P., Leturque, A., Burnol, A. F., Penicaud, L. & Girard, J. A method to quantify glucose utilization in vivo in skeletal muscle and white adipose tissue of the anaesthetized rat. *Biochem J* **228**, 103-110, doi:10.1042/bj2280103 (1985).

76 Martianova, E., Aronson, S. & Proulx, C. D. Multi-Fiber Photometry to Record Neural Activity in Freely-Moving Animals. *J Vis Exp*, doi:10.3791/60278 (2019).

77 Suyama, S. *et al.* Plasticity of calcium-permeable AMPA glutamate receptors in Pro-opiomelanocortin neurons. *Elife* **6**, doi:10.7554/eLife.25755 (2017).

78 Cavalieri, R. *et al.* Activating ligands of Uncoupling protein 1 identified by rapid membrane protein thermostability shift analysis. *Mol Metab* **62**, 101526, doi:10.1016/j.molmet.2022.101526 (2022).

79 Desousa, B. R. *et al.* Calculation of ATP production rates using the Seahorse XF Analyzer. *EMBO Rep* **24**, e56380, doi:10.15252/embr.202256380 (2023).
