# Supplementary material for: GLP-1R–GIPR–PPARα/γ/δ quintuple agonism corrects obesity and diabetes in mice
Source: Nature. 2026 Apr 29;653(8115):776–85. doi: 10.1038/s41586-026-10427-5 (PMC13190304; doi:10.1038/s41586-026-10427-5)
Supplement: Supplementary file 2 — Reporting Summary [file 41586_2026_10427_MOESM2_ESM.pdf]

nature portfolio

Corresponding author(s): Timo Müller

Last updated by author(s): Feb 14, 2026

Reporting Summary

Nature Portfolio wishes to improve the reproducibility of the work that we publish. This form provides structure for consistency and transparency in reporting. For further information on Nature Portfolio policies, see our Editorial Policies and the Editorial Policy Checklist.

Statistics

For all statistical analyses, confirm that the following items are present in the figure legend, table legend, main text, or Methods section.

n/a	Confirmed	
☐	☒	The exact sample size (*n*) for each experimental group/condition, given as a discrete number and unit of measurement
☐	☒	A statement on whether measurements were taken from distinct samples or whether the same sample was measured repeatedly
☐	☒	The statistical test(s) used AND whether they are one- or two-sided<br>*Only common tests should be described solely by name; describe more complex techniques in the Methods section.*
☐	☒	A description of all covariates tested
☐	☒	A description of any assumptions or corrections, such as tests of normality and adjustment for multiple comparisons
☐	☒	A full description of the statistical parameters including central tendency (e.g. means) or other basic estimates (e.g. regression coefficient) AND variation (e.g. standard deviation) or associated estimates of uncertainty (e.g. confidence intervals)
☐	☒	For null hypothesis testing, the test statistic (e.g. *F*, *t*, *r*) with confidence intervals, effect sizes, degrees of freedom and *P* value noted<br>*Give P values as exact values whenever suitable.*
☒	☐	For Bayesian analysis, information on the choice of priors and Markov chain Monte Carlo settings
☒	☐	For hierarchical and complex designs, identification of the appropriate level for tests and full reporting of outcomes
☐	☒	Estimates of effect sizes (e.g. Cohen's *d*, Pearson's *r*), indicating how they were calculated

Our web collection on statistics for biologists contains articles on many of the points above.

Software and code

Policy information about availability of computer code

Data collection	Indirect calorimetry data were collected using Promethion Live (version 24.9.1, Sable Systems International). qPCR data were acquired using QuantStudio (version 1.3, Thermo Fisher Scientific). BRET and ELISA data were acquired using PheraStar (version 4.00 R4, BMG Labtech). FASTQ files were generated using Illumina bcl2fastq Conversion Software (version 2.17.1.14). Brainstem cFOS images were acquired using LAS X (version 3.5.7.23225, Leica Microsystems); hypothalamic cFOS images were obtained by z-stack scanning with AxioScan 7 (ZEN Blue version 3.5, Zeiss). Proteomic data were processed using Spectronaut (v.19.5). For analysis of bulk RNA sequencing data, FASTQ files were generated from base calls using bcl2fastq (v. 2.17.1.14). Quality control for the raw transcriptomics sequencing reads was done using FastQC (v. 0.12.0). Transcript counts were imported into python using scanpy and decoupler (v. 2.14.0). Gene expression z-scores of differentially expressed genes were plotted using Matplotlib (doi.org/10.5281/zenodo.592536), ggplot2 (v.3.5.2) and seaborn (0.13.2). Fiber photometric data were aquired using Synapse (v. 102). Cardiac function was assessed using the Vevo 3100 High-Resolution Micro-Ultrasound Imaging System (FUJIFILM VisualSonics, Toronto, Canada). Blood pressure was measured using the CODA® Monitor tail-cuff system (AD Instruments, Oxford, United Kingdom). Calcium indicator signals from POMC neurons were measured using the LUX RZ10X processor (Tucker-Davis Technologies). Whole-cell current clamp was performed with a Multiclamp 700A amplifier (Molecular Devices, CA). Cultured adipocytes were imaged using Invitrogen™ EVOS™ XL Core Imaging System (Thermo Fisher Scientific). Immortalized mouse brown preadipocytes were analyzed using the XF96 Seahorse extracellular flux analyzer (Agilent Technologies).
Data analysis	Statistical analyses were performed using GraphPad Prism (v.10.03) and SPSS (v.31, IBM). cFOS data were analyzed using QuPath (version 0.4.4, University of Edinburgh). Raw proteomics data were processed with Spectronaut (version 19.5, Biognosys). Imputation and data analysis were performed in Perseus (version 1.6.15.0, Max Planck Institute of Biochemistry). Histological analyses were performed using Visiopharm (version 2018.9, Visiopharm A/S). FastQC (version 0.12.0) was used for quality control of sequencing reads. STAR (v. 2.7.10) was used for alignment to the GRCm38 reference genome. Transcript counts were analyzed in Python (version 3.13) using scanpy (version 1.11.1) and decoupler (version 2.14.0). Gene expression normalization was performed with pyDESeq2 (version 0.5.0). Differential expression analysis used

the Wald test. Data visualization was conducted with Matplotlib (version 3.10.1), ggplot2 (version 3.5.2), and seaborn (version 0.13.2). Functional enrichment analysis was performed using Over Representation Analysis (ORA) via decoupler. Ejection fraction (EF), fractional shortening (FS), cardiac output (CO), stroke volume (SV) and heart rate (HR) were quantified with VevoLab (v.5.10.0). Blood pressure was calculated with LabChart (v. 8.1.24). Electrophysiological data were sampled with an Apple Macintosh computer using AxoGraph X (v. 1.7.3). AP frequency was plotted with Igor Pro (v.9) and Prism (v. 10.0.3). Cultured adipocytes were analysed using ImageJ (v. 1.54)

For manuscripts utilizing custom algorithms or software that are central to the research but not yet described in published literature, software must be made available to editors and reviewers. We strongly encourage code deposition in a community repository (e.g. GitHub). See the Nature Portfolio guidelines for submitting code & software for further information.

Data

Policy information about availability of data

All manuscripts must include a data availability statement. This statement should provide the following information, where applicable:

- Accession codes, unique identifiers, or web links for publicly available datasets
- A description of any restrictions on data availability
- For clinical datasets or third party data, please ensure that the statement adheres to our policy

Raw data for the proteomic and transcriptomic analysis are available via Pride (PXD062990). Raw data from the bulk RNAseq are available in the GEO under SuperSeries accession number GSE314029. All data used for the statistical analysis are available in the Data Source File, along with the GraphPad Prism-derived report on the statistical analysis. The statistical report contains the mean difference between the treatment groups, the 95% confidence intervals, the significance summary, and the exact p-values (unless p<0.0001). A summary of the statistical tests are along the p-values for the main treatment effects also shown in Supplementary Table 1. Transcripts were aligned using the GRCm38 reference genome (GenBank accession GCA_000001635.20).

Research involving human participants, their data, or biological material

Policy information about studies with human participants or human data. See also policy information about sex, gender (identity/presentation), and sexual orientation and race, ethnicity and racism.

Reporting on sex and gender	n/a
Reporting on race, ethnicity, or other socially relevant groupings	n/a
Population characteristics	n/a
Recruitment	n/a
Ethics oversight	n/a

Note that full information on the approval of the study protocol must also be provided in the manuscript.

Field-specific reporting

Please select the one below that is the best fit for your research. If you are not sure, read the appropriate sections before making your selection.

☒ Life sciences ☐ Behavioural & social sciences ☐ Ecological, evolutionary & environmental sciences

For a reference copy of the document with all sections, see nature.com/documents/nr-reporting-summary-flat.pdf

Life sciences study design

All studies must disclose on these points even when the disclosure is negative.

Sample size	For animal studies, sample sizes were calculated based on a power analysis assuming that a greater or equal (>/=) 5 g difference in body weight between genotypes can be assessed with a power of >/= 75% when using a 2-sided statistical test under the assumption of a standard deviation of 3.5 and an alpha level of 0.05. For in vitro and ex vivo experiments, sample sizes were estimated based on our experience using the same technologies in the same cell system (PMID: 37277609, PMID: 35995995, PMID: 33556643, PMID: 40204014) or animal model (PMID: 38418586, PMID: 37946085).
Data exclusions	No data were excluded from the analysis unless scientific (e.g. significant outlier identified by the Grubbs test for outlier) or animal welfare reasons (e.g. injury due to fighting) demanded exclusion. Outliers are stated in the data source file. Four samples were excluded from the Protemic analysis (one per tissue: Quad, brain stem, eWAT, and hypothalamus) as they did not pass quality control.
Replication	All reported in vivo, ex vivo and in vitro data correspond to independent biological replicates. The exact samples sizes are reported in the figure legends.
Randomization	Animals were either randomly assigned into treatment groups, or were grouped based on their genotype (WT or KO). At study start, only age-matched mice were included in the studies. There were no other covariats controlled.

Blinding	For in vivo studies, drugs were aliquoted by a lead scientist in number-coded vials and most, but not all, handling investigators were blinded to the treatment condition. Analyses of glucose and insulin tolerance were performed by experienced research assistants who did not know prior treatment conditions. Studies on drug effects on the cardiovascular system were performed blinded, with only the main PI but not the handling investigators being aware of the treatment groups. Ex vivo and in vitro studies were performed in ID coded vials, and with most, but not all investigators, being blinded to the underlying genotypes and treatment conditions.

Reporting for specific materials, systems and methods

We require information from authors about some types of materials, experimental systems and methods used in many studies. Here, indicate whether each material, system or method listed is relevant to your study. If you are not sure if a list item applies to your research, read the appropriate section before selecting a response.

Materials & experimental systems

n/a	Involved in the study
☐	☒ Antibodies
☐	☒ Eukaryotic cell lines
☒	☐ Palaeontology and archaeology
☐	☒ Animals and other organisms
☒	☐ Clinical data
☒	☐ Dual use research of concern
☒	☐ Plants

Methods

n/a	Involved in the study
☒	☐ ChIP-seq
☒	☐ Flow cytometry
☒	☐ MRI-based neuroimaging

Antibodies

Antibodies used	cFos (#MA5-15055, Invitrogen, 1:400), anti-rabbit Alexa546 (#A10040, Invitrogen, Karlsruhe, Germany, 1:2,000), rabbit anti-insulin (#3014, Cell Signaling Technology, Danvers, USA, 1:800), mouse anti glucagon (#G2654, Merck, Darmstadt, Germany, 1:1000,), goat anti-rabbit AF750 (#A21039, Invitrogen, Karlsruhe, Germany, 1:100), donkey anti-mouse AF555 (#A32773, Invitrogen, Karlsruhe, Germany, 1:200)
Validation	cFOS (Invitrogen, #MA5-15055): The cFOS monoclonal antibody Invitrogen #MA5-15055 was verified by Relative expression to ensure that the antibody binds to the antigen stated. The antibody shows reactivity in bovine, hamster, human, mouse, pig and rat. The antibody can be used for western blot, immunhistochemistry, immuncytochemistry, flow cytometry and ChIP assays. The antibody does not cross-react with other Fos proteins, including FosB, FRA1 and FRA2. Immunofluorescence analysis of c-Fos was performed using 70% confluent log phase HeLa cells treated with 200 ng/mL EGF for 30 min. The cells were fixed with 4% paraformaldehyde for 10 minutes, permeabilized with 0.1% Triton™ X-100 for 10 minutes, and blocked with 1% BSA for 1 hour at room temperature. The cells were labeled with c-Fos Monoclonal Antibody (T.142.5) (product # MA5-15055) at 1:250 dilution in 0.1% BSA, incubated overnight at 4 degree Celsius and then labeled with Goat anti-Rabbit IgG (H+L) Superclonal™<br><br>Anti-rabbit Alexa546 (#A10040, Invitrogen, Karlsruhe, Germany): The donkey anti-Rabbit IgG (H+L) Highly Cross-Adsorbed Secondary Antibody, Alexa Fluor™ 546 secondary antibody was verified using HepG2 cells stained with alpha-1 antitrypsin Rabbit Polyclonal Primary Antibody (Product # PA5-16661). The antibody was used at a concentration of 4 µg/mL in phosphate buffered saline containing 0.2 % BSA for 45 minutes at room temperature, for detection of alpha-1 antitrypsin in the cytoplasm. Nuclei were stained with DAPI in SlowFade® Gold Antifade Mountant. F-actin was stained with Alexa Fluor® 488 Phalloidin. No nonspecific staining was observed with the secondary antibody alone or with an isotype control. The highly specific antibody has been used in n=63 scientific publications.<br><br>Rabbit anti-insulin (#3014, Cell Signaling Technology, Danvers, USA): The Insulin (C27C9) Rabbit Monoclonal Antibody #3014 has been used in n=156 scientific publications. The specificity of this antibody has been verified using western blot and immunhistochemical analysis in paraffin-embedded mouse pancreata. Western blot analysis of lysates (1.0 mg/mL) from INS-1 cells using Insulin (C27C9) Rabbit mAb #3014 show a single band at the expected size. The virtual lane shows the target band at 1:10 and 1:50 dilutions of primary antibody. The corresponding electropherogram plots chemiluminescence by molecular weight along the capillary at 1:10 and 1:50 dilutions of primary antibody. The experiment was performed under reducing conditions on the JessTM Simple Western instrument from ProteinSimple, a BioTechne brand, using the 12-230 kDa separation module.<br><br>Mouse anti glucagon (#G2654, Merck, Darmstadt, Germany, 1:1000): This monoclonal Anti-Glucagon antibody produced in mouse has been verified for immunostaining of pancreatic tissue in flow cytometry and immunofluorescence imaging of pancreas cells for immunohistochemistry and morphology of pancreas. The antibody reacts specifically against pancreatic glucagon and exhibits only very weak cross-reaction with gut glucagon (enteroglucagon). The antibody may be used for the immunohistochemical staining of Bouin's-fixed, and formalin-fixed, paraffin-embedded pancreatic tissue sections. Binds to glucagon with an affinity constant of 6.1 × 108 M-1 in RIA.Monoclonal anti-Glucagon antibody can be used as an analytical tool for quantification of the hormone. It can also be used for immunocytochemical staining of formalin fixed and Bouin-fixed, paraffin-embedded pancreatic tissue sections. Mouse anti-Glucagon antibody reacts specifically with pancreatic glucagon. The product has also shown cross reactivity with glucagon-containing cells in fixed sections of pancreas from dog, mouse, rat, rabbit, porcine, guinea pig, cat and human and weak cross reactivity for gut glucagon (enteroglucagon). Monoclonal Anti-Glucagon reacts with pancreatic glucagon in RIA and immunocytochemistry. The affinity constant of 6.1 x 10(8) L/M in RIA. The antibody weakly cross-reacts with gut glucagon (enteroglucagon) in an immunohistological assay. Cross-reactivity has been observed with glucagon-containing cells in fixed sections of pancreas from human, porcine, dog, rabbit, mouse, rat, guinea pig, and cat.

Goat anti-rabbit AF750 (#A21039, Invitrogen, Karlsruhe, Germany, 1:100): Goat anti-Rabbit IgG (H+L) Cross-Adsorbed Secondary Antibody, Alexa Fluor™ 750 has been used in 123 scientific manuscripts. Verification included immunofluorescence analysis of Goat anti-Rabbit IgG (H+L) Cross-Adsorbed Secondary Antibody, Alexa Fluor™ 750 (Product # A-21039) using MCF 10A (positive model) and T-47D (negative model) cells stained with Vimentin Polyclonal Antibody (Product # PA5-27231). The cells were fixed with 4% paraformaldehyde for 10 minutes, permeabilized with 0.1% Triton™ X-100 for 10 minutes, blocked with 1% BSA for 1 hour and labeled with 2 µg/mL primary antibody for 3 hours at room temperature. Goat anti-Rabbit IgG (H+L) Cross-Adsorbed Secondary Antibody, Alexa Fluor™ 750 (Product # A-21039, 1:2000 dilution) in 0.1% BSA in PBS for 45 minutes at room temperature, was used for detection of Vimentin in the cytoplasm. Nuclei were stained with Hoechst33342 (Product # H1399). F-actin was stained with Alexa Fluor® 488 Phalloidin (Product # A12379, 1:300). The specificity of the secondary antibody was proved by the absence of signal in T-47D (negative model for vimentin) due to no primary antibody binding. Nonspecific staining was not observed with secondary antibody alone. The images were captured at 40X magnification in CellInsight CX7 LZR High-Content Screening (HCS) Platform (Product # CX7A1110LZR) and externally deconvoluted.

Donkey anti-mouse AF555 (#A32773, Invitrogen, Karlsruhe, Germany, 1:200): Donkey anti-Mouse IgG (H+L) Highly Cross-Adsorbed Secondary Antibody, Alexa Fluor™ Plus 555 has been used in 182 scientific manuscripts. Verification of the antibody includes immunofluorescent analysis of tubulin in A549 cells. The cells were fixed with 4% formaldehyde for 20 mins, permeabilized with 0.5% Triton X-100 in PBS for 20 mins, washed 3X in PBS and blocked with 3% BSA in PBS for 30 mins at RT. Cells were stained with a tubulin antibody at a dilution of 1:2000 in 3% BSA in PBS for 1 hr at RT, washed 3X in PBS and then incubated with Invitrogen Alexa Fluor Plus 555 donkey anti-mouse IgG secondary antibody (Product # A32773) prepared in 3% BSA in PBS at a dilution of 1:1000 for 1 hr at RT in the presence of NucBlue Live ReadyProbes Reagent (Product # R37605). The image contains overlay of tubulin and nuclei. Images were taken on an EVOS FL Auto 2 Imaging System (Product # AMAFD2000) with an Olympus 40X Super Apochromat objective (Product # AMEP4754) at 40X magnification. Actin was stained using Alexa Fluor Plus Phalloidin (Product # A30105).

Eukaryotic cell lines

Policy information about cell lines and Sex and Gender in Research

Cell line source(s)	HEK293T cells (ATCC, USA)
Authentication	Authentication according to the manufacturer's website: The 293T cell line, originally referred as 293tsA1609neo, is a highly transfectable derivative of human embryonic kidney 293 cells, and contains the SV40 T-antigen.This cell line is competent to replicate vectors carrying the SV40 region of replication. It gives high titers when used to produce retroviruses. It has been widely used for retroviral production, gene expression and protein production. Product related references include DuBridge et al., Mol Cell Biol. 1987 Jan;7(1):379-87 and Pear et al., Proc Natl Acad Sci U S A. 1993 Sep 15;90(18):8392-6. https://www.lgcstandards-atcc.org/Products/All/CRL-3216.aspx?geo_country=de#generalinformation
Mycoplasma contamination	cell lines were free of mycoplasm contaminations
Commonly misidentified lines (See ICLAC register)	no misidentified cell lines were used in the manuscript

Animals and other research organisms

Policy information about studies involving animals; ARRIVE guidelines recommended for reporting animal research, and Sex and Gender in Research

Laboratory animals

Figure 1a,b: 43 wk old male DIO wildtype C57BL6/J mice
Figure 1c-e: 45 wk old male DIO wildtype C57BL6/J mice
Figure 1h: 28-34 wk old male wildtype C57BL6/J mice
Figure 1l: 43 wk old male DIO wildtype C57BL6/J mice
Figure 1m: 45 wk old male DIO wildtype C57BL6/J mice
Figure 1n: 43 wk old male DIO wildtype C57BL6/J mice
Figure 1o: 45 wk old male DIO wildtype C57BL6/J mice
Figure 2a: 33 wk old male DIO wildtype C57BL6/J mice
Figure 2b: 35 wk old male DIO wildtype C57BL6/J mice
Figure 2c: 33 wk old male DIO wildtype C57BL6/J mice
Figure 2d-f: 35 wk old male DIO wildtype C57BL6/J mice
Figure 2g-j: 28-30 wk old male DIO wildtype C57BL6/J mice
Figure 2k-m: 55 wk old male DIO wildtype C57BL6/J mice
Figure 2n,o: 35 wk old male DIO C57BL6/J mice
Figure 2p,q: 8 wk old male chow fed C57BL6/J mice
Figure 2r-v: 27wk old male DIO C57BL6/J mice
Figure 3a: 40 wk old male DIO wildtype C57BL6/J mice
Figure 3b: 45 wk old male DIO wildtype C57BL6/J mice
Figure 3c: 40 wk old male DIO wildtype C57BL6/J mice
Figure 3d: 42 wk old male DIO wildtype C57BL6/J mice
Figure 3e: 45 wk old male DIO wildtype C57BL6/J mice
Figure 3f,g: 43 wk old male DIO wildtype C57BL6/J mice
Figure 3h-m: 45 wk old male DIO wildtype C57BL6/J mice
Figure 3n,o: 51 wk old male DIO wildtype C57BL6/J mice
Figure 3o: 47 wk (Day 0) and 51 (Day 26) wk old male DIO wildtype C57BL6/J mice
Figure 3p: 51 wk old male DIO wildtype C57BL6/J mice

Figure 3q-v: 20 wk old male DIO wildtype C57BL6/J mice
Figure 4a,b: 14-24 wk old male C57BL6/J mice wildtype or Vglut2 Cre GLP-1R KO mice
Figure 4c: 39 wk old male C57BL6/J DIO wildtype and global Gipr KO mice
Figure 4d,e: 68 wk old male DIO wildtype C57BL6/J mice
Figure 4f: 70 wk old male DIO wildtype C57BL6/J mice
Figure 4g: 56 wk old male DIO wildtype C57BL6/J mice
Figure 4h: 55 wk old male DIO wildtype C57BL6/J mice
Figure 4i,j: 32 wk old male DIO wildtype or double incretin receptor KO C57BL6/J mice
Figure 4k-m: 33 wk old male DIO wildtype or double incretin receptor KO C57BL6/J mice
Figure 5a-f: 45 wk old male DIO C57BL6/J mice
Figure 5h-m: 32 wk old male DIO C57BL6/J mice
Figure 5n,o: Male chow fed Pomc-cre)16Lowl/J mice (N=5, 10-17 wk old; N=1, 30 wk old)
Figure 5p: 8-10 wk old male chow fed C57BL6/J POMC-GFP mice
EDF2c-h: 45 wk old male DIO wildtype C57BL6/J mice
EDF3a: 27 wk old male DIO wildtype C57BL6/J mice
EDF3b: 28 wk old male DIO wildtype C57BL6/J mice
EDF3c: 27 wk old male DIO wildtype C57BL6/J mice
EDF3d,e: 47 wk old male DIO C57BL6/J mice
EDF3f-i: 49 wk old male DIO C57BL6/J mice
EDF3j-l: 33 wk old male DIO wildtype C57BL6/J mice
EDF4a-l: 35 wk old male DIO C57BL6/J mice
EDF5a-d: 35 wk old male DIO wildtype C57BL6/J mice
EDF5f-s: Immortalized BAT cells were obtained from 6-8 wk old chow-fed female C57BL6/J mice
EDF6a: 10 wk old male C57BL6/J db/db mice
EDF6b: 13 wk old male C57BL6/J db/db mice
EDF6c: 10 wk old male C57BL6/J db/db mice
EDF6d: 11 wk old male C57BL6/J db/db mice
EDF6e: 12 wk old male C57BL6/J db/db mice
EDF6f-h: 13 wk old male C57BL6/J db/db mice
EDF6i: 14-24 wk old male C57BL6/J wildtype and Vglut2-Cre GLP-1R flx/flx mice
EDF6j: 24 wk old male wildtype C57BL6/J mice
EDF6k: 26 wk old male wildtype C57BL6/J mice
EDF6j: 24 wk old male wildtype C57BL6/J mice
EDF6k: 26 wk old male wildtype C57BL6/J mice
EDF7a: 47 wk old male DIO wildtype C57BL6/J mice
EDF7b-e: 49 wk old male DIO wildtype C57BL6/J mice
EDF7c: 47 wk old male DIO wildtype C57BL6/J mice
EDF7g: 31 wk old male DIO wildtype C57BL6/J mice
EDF7e: 32 wk old male DIO wildtype C57BL6/J mice
EDF8a-p: 32 wk old male DIO C57BL6/J mice
EDF9a,b: 32 wk old male DIO C57BL6/J mice

Wild animals	No wild animals were used in the study
Reporting on sex	All studies were performed in male mice, since female mice are largely resistant to the development of diet induced obesity and the development of high-fat diet induced insulin resistance.
Field-collected samples	No field collected samples were used in the study
Ethics oversight	Experiments were performed in accordance with the Animal Protection Law of the European Union after permission by the Governments of Upper Bavaria, Germany, or Copenhagen, Denmark, or by the Institutional Animal Care and Use Committees of the Universities of Texas Southwestern, Michigan, Duke or Yale, USA.

Note that full information on the approval of the study protocol must also be provided in the manuscript.

Plants

Seed stocks	n/a
Novel plant genotypes	n/a
Authentication	n/a
