# Supplementary material for: GLP-1R–GIPR–PPARα/γ/δ quintuple agonism corrects obesity and diabetes in mice
Source: Nature. 2026 Apr 29;653(8115):776–85. doi: 10.1038/s41586-026-10427-5 (PMC13190304; doi:10.1038/s41586-026-10427-5)

Original Pictures for Figure 2p

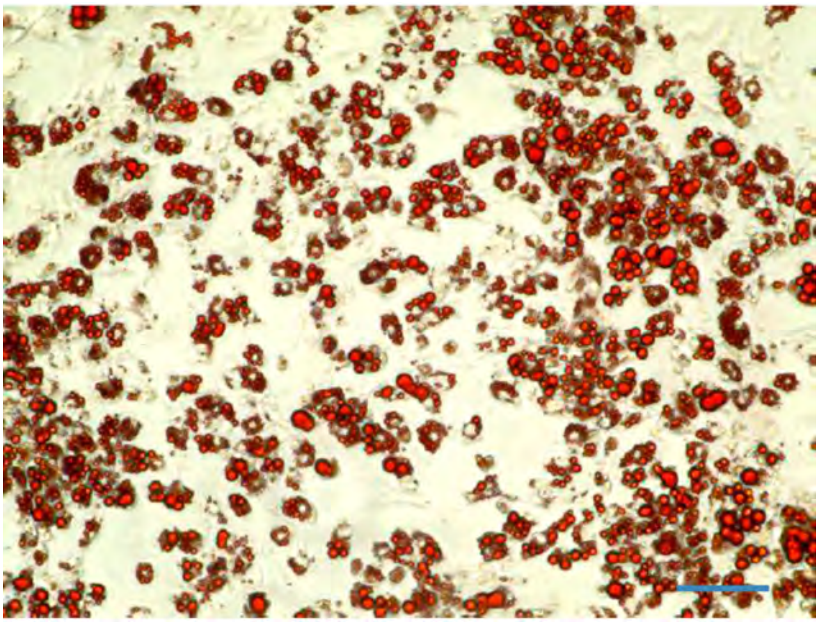

GLP-1:GIP #1

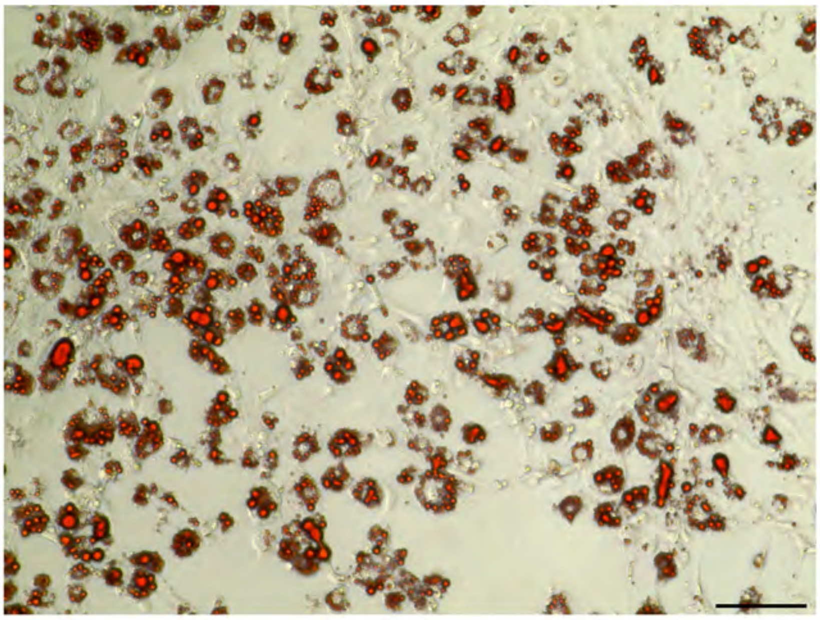

GLP-1:GIP #2

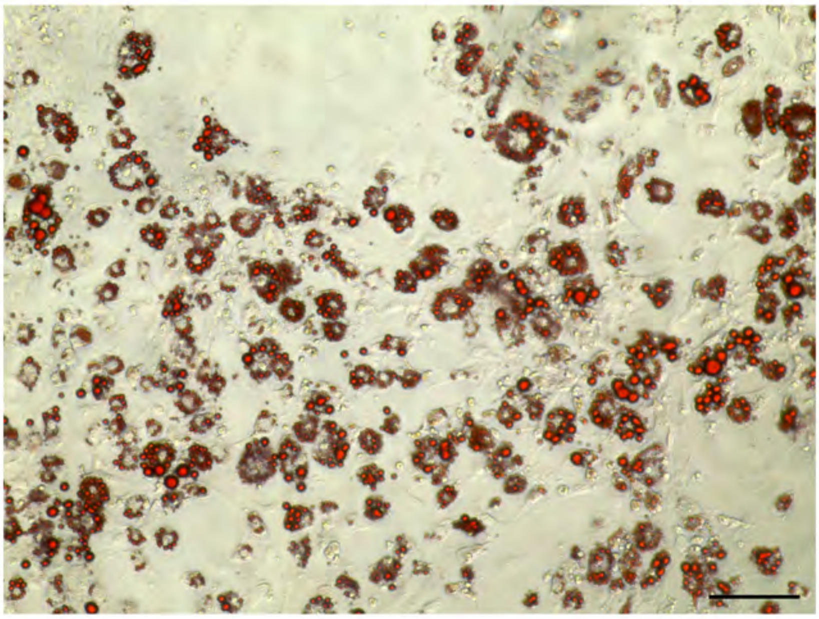

GLP-1:GIP #3

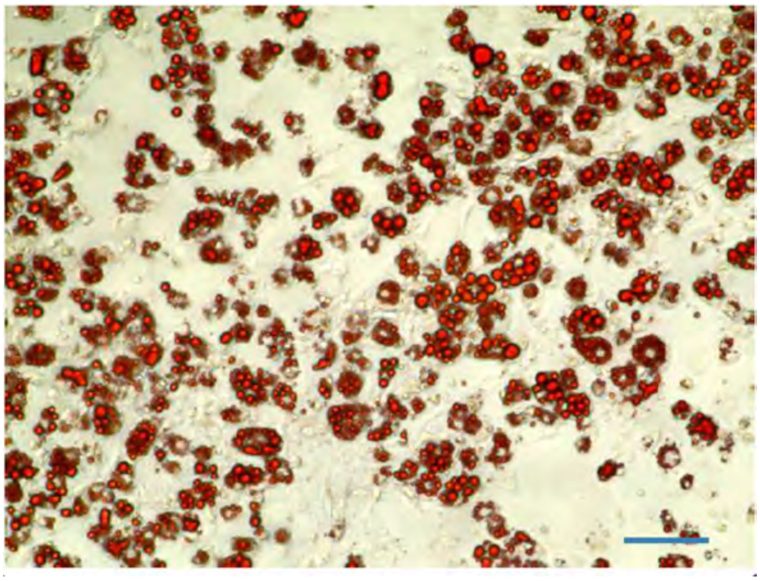

GLP-1:GIP:lani #1

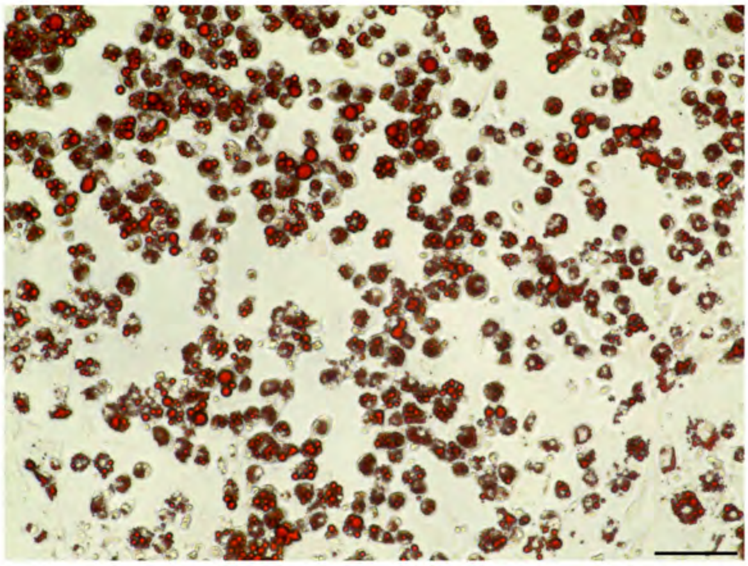

GLP-1:GIP:lani #2

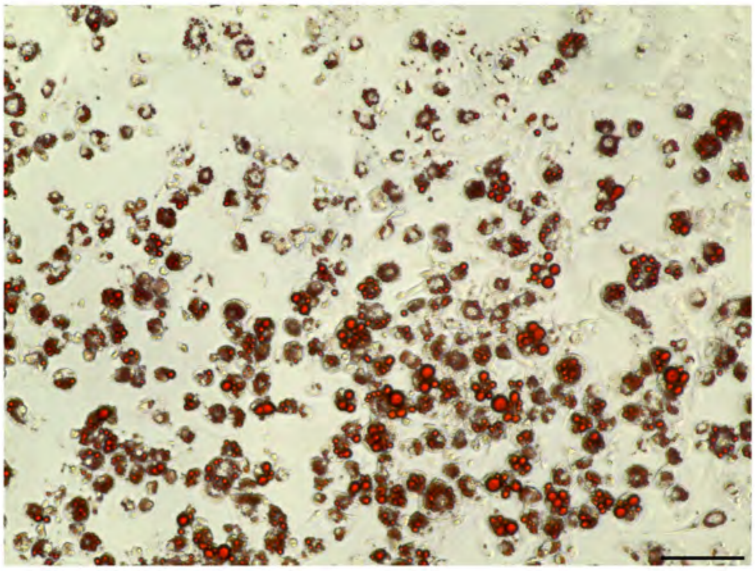

GLP-1:GIP:lani #3

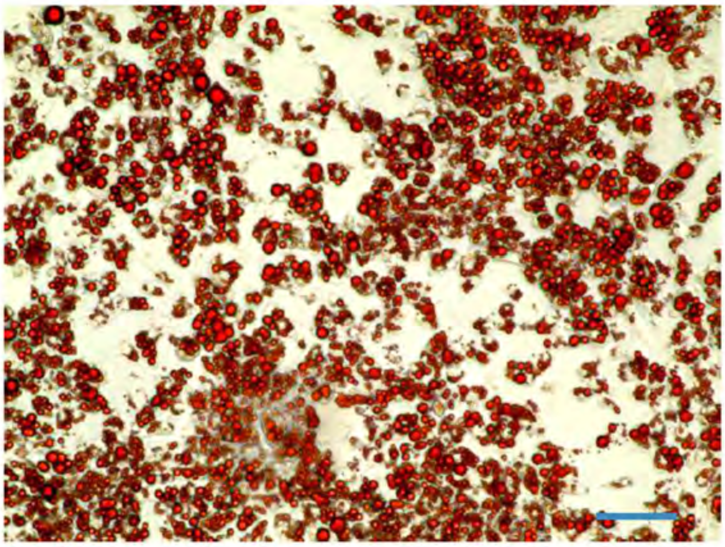

Rosi #1

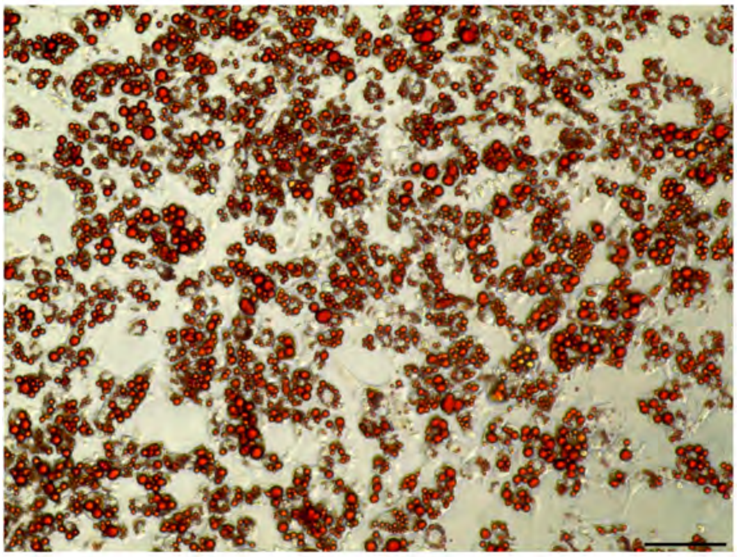

Rosi #2

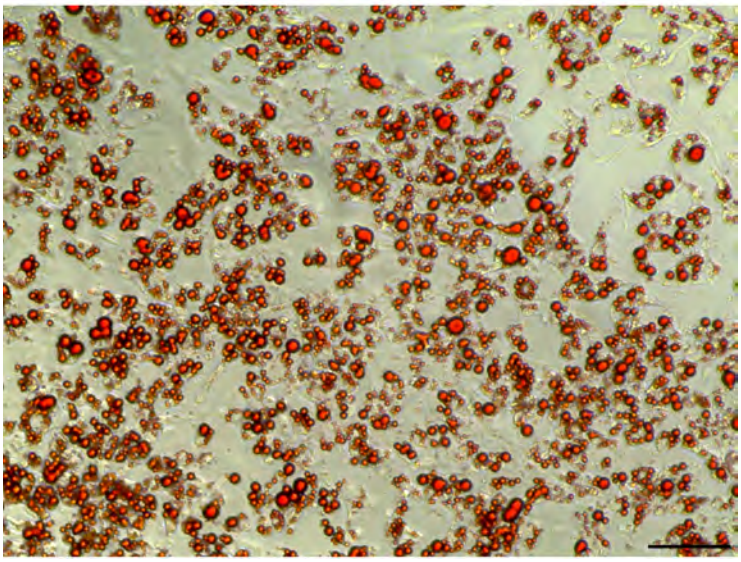

Rosi #3

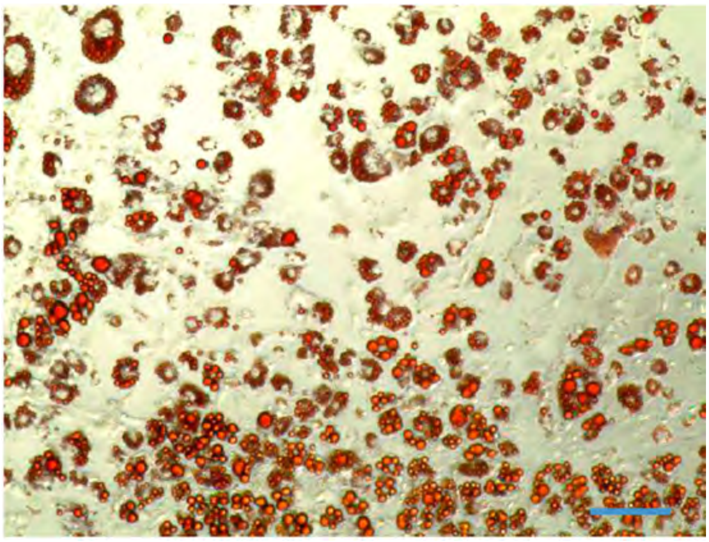

Vhcl #1

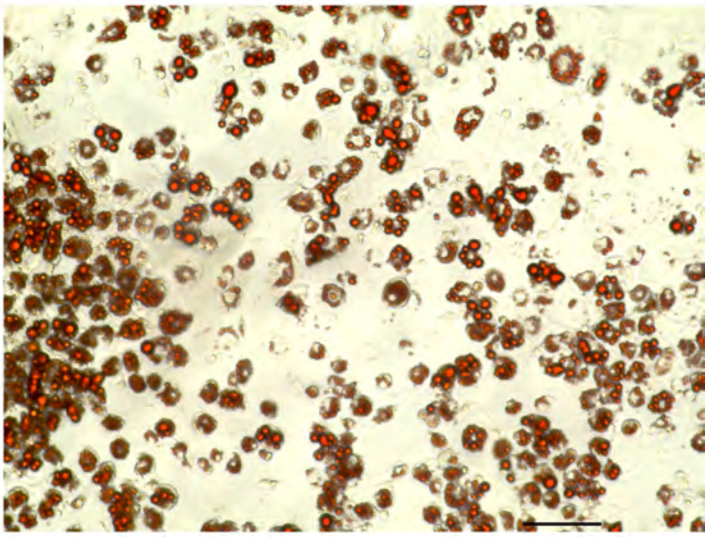

Vhcl #2

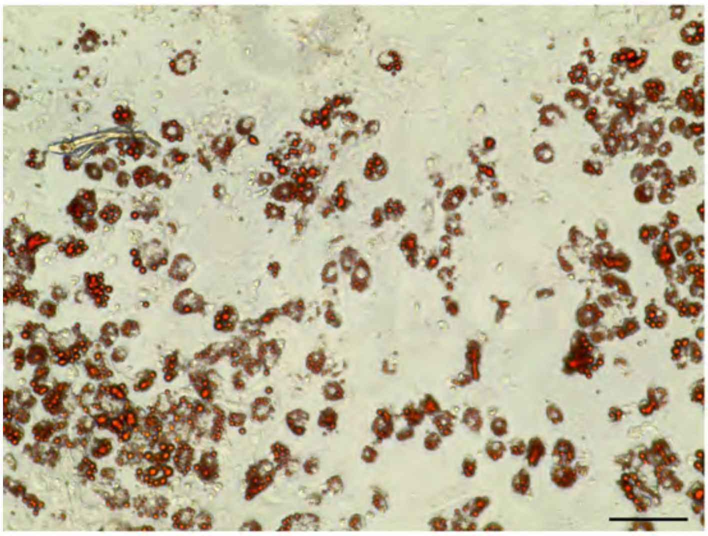

Vhcl #3

Original Pictures for Figure 3l

eWAT

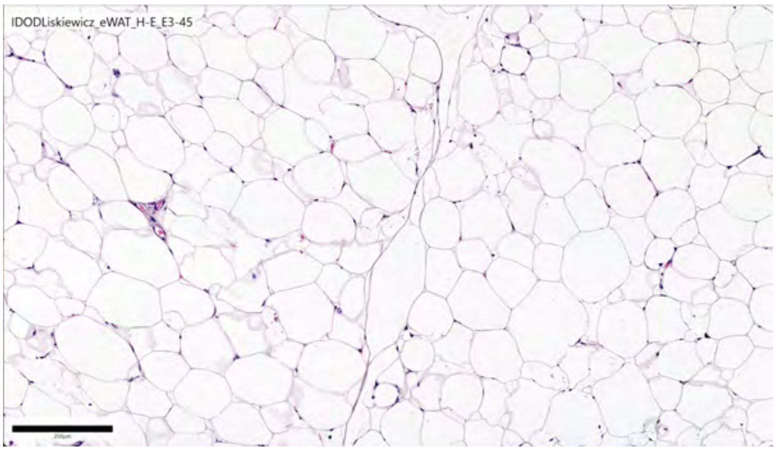

Co-Therapy #1

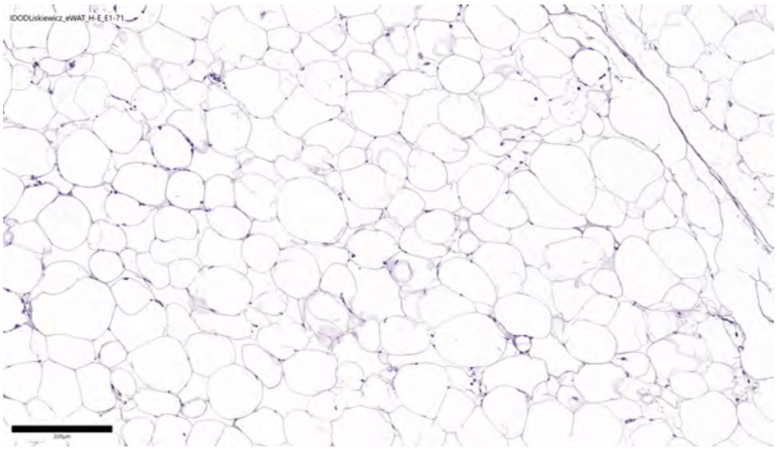

Co-Therapy #2

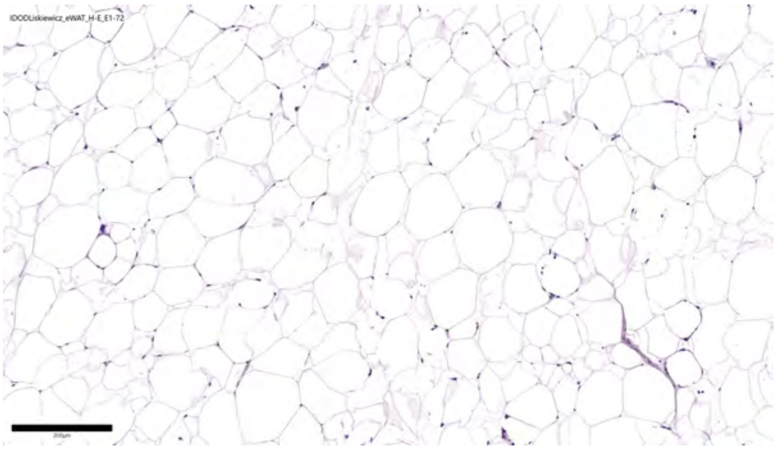

Co-Therapy #3

Original Pictures for Figure 3l

eWAT

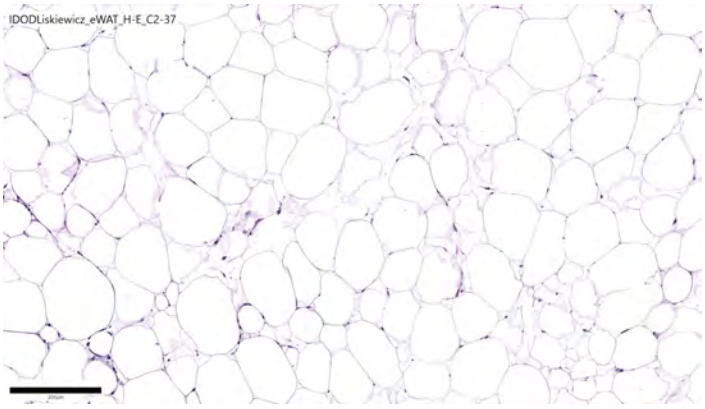

Conjugate #1

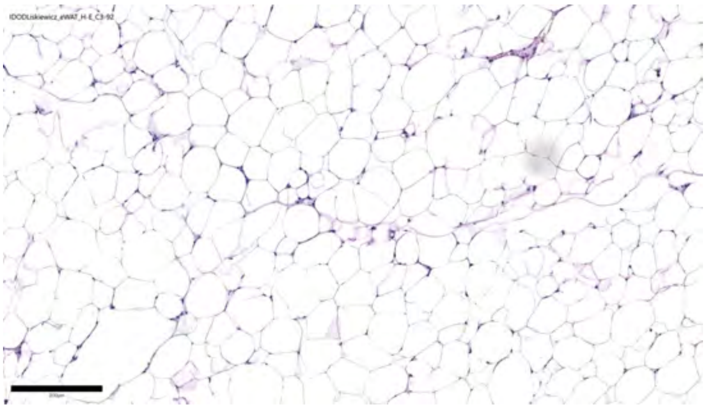

Conjugate #2

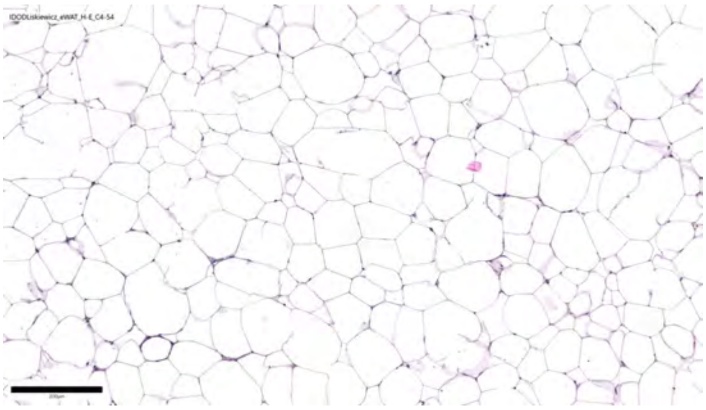

Conjugate #3

Original Pictures for Figure 3l

eWAT

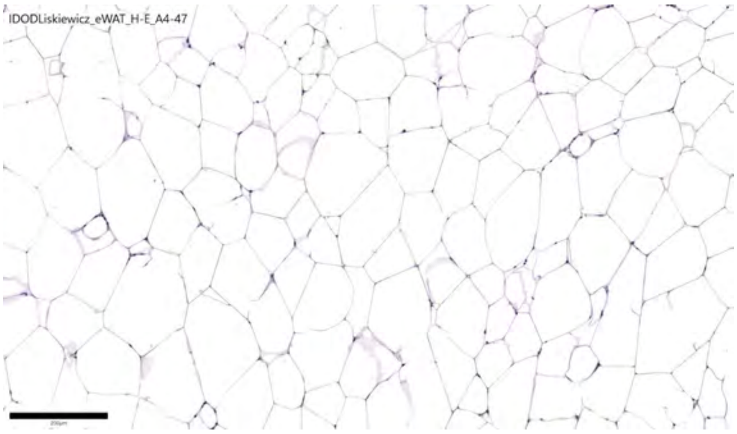

Vhcl #1

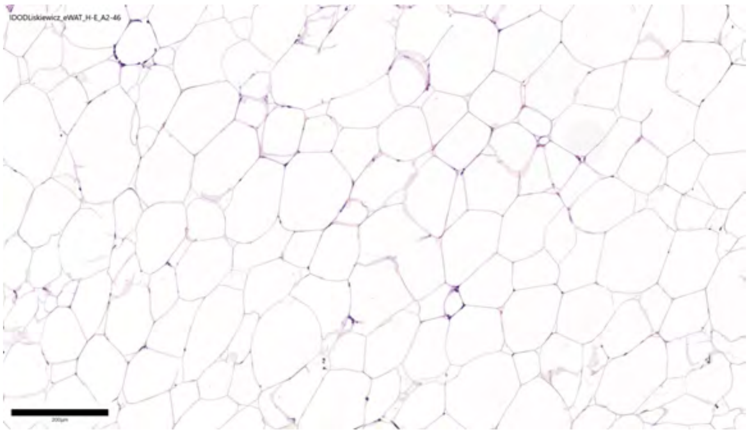

Vhcl #2

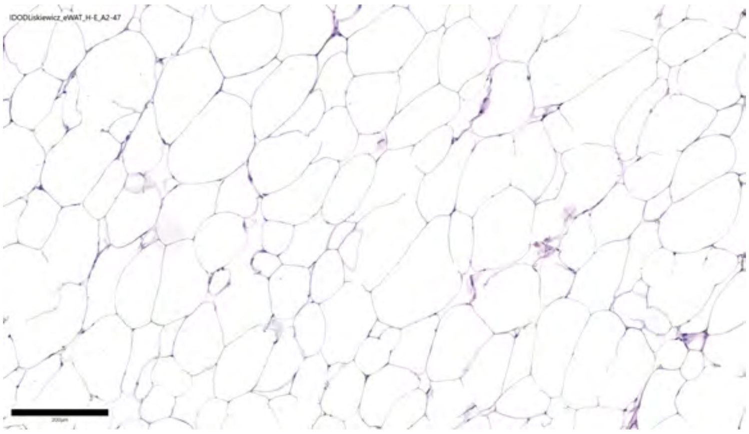

Vhcl #3

Original Pictures for Figure 3l

heart

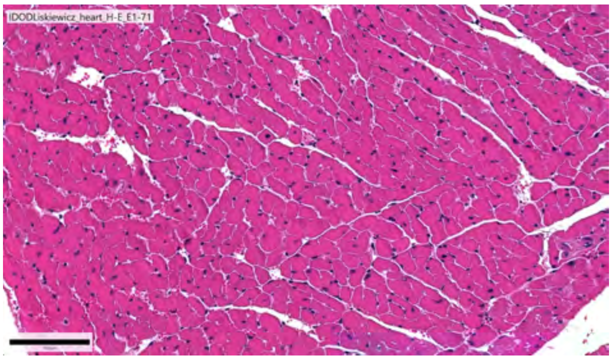

Co-Therapy #1

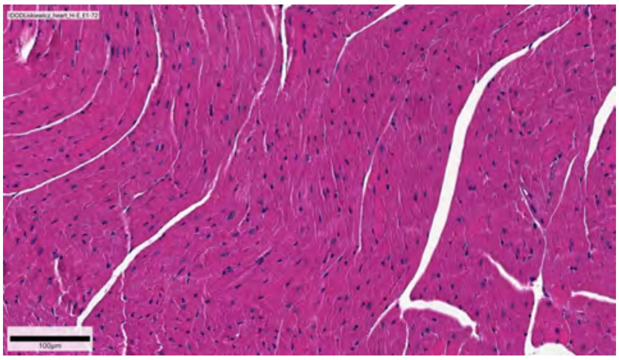

Co-Therapy #2

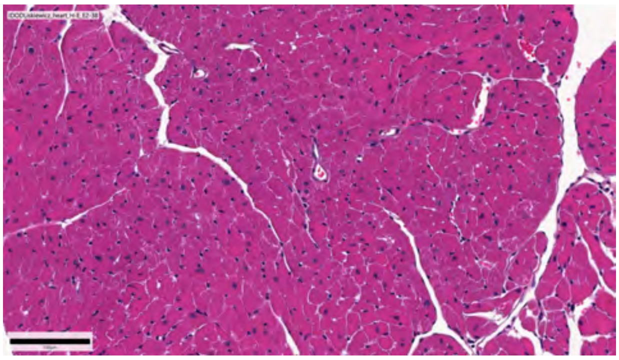

Co-Therapy #3

Original Pictures for Figure 3l

Heart

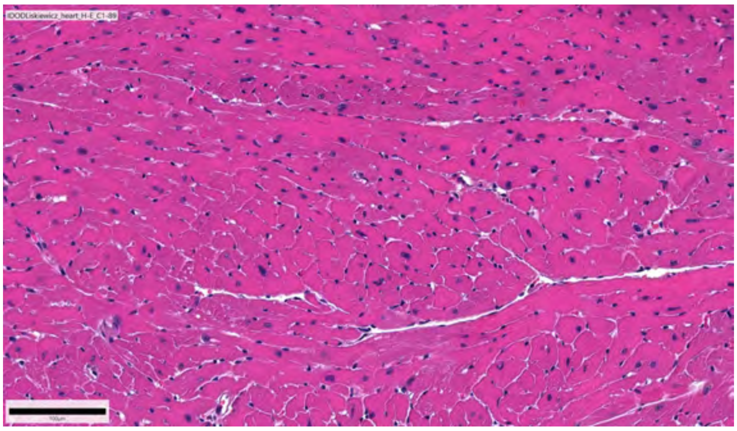

Conjugate #1

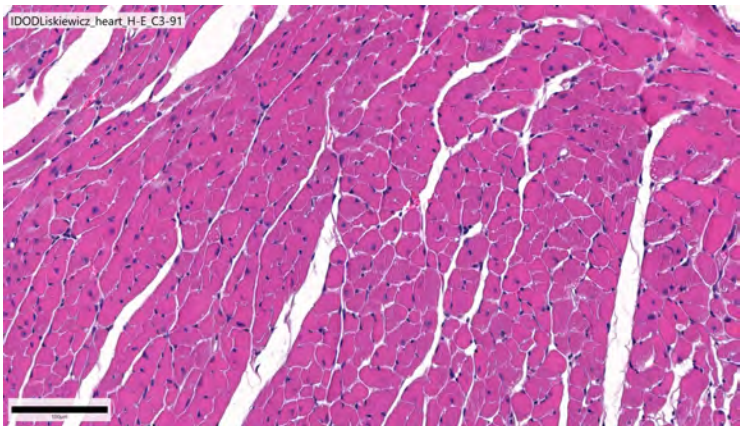

Conjugate #2

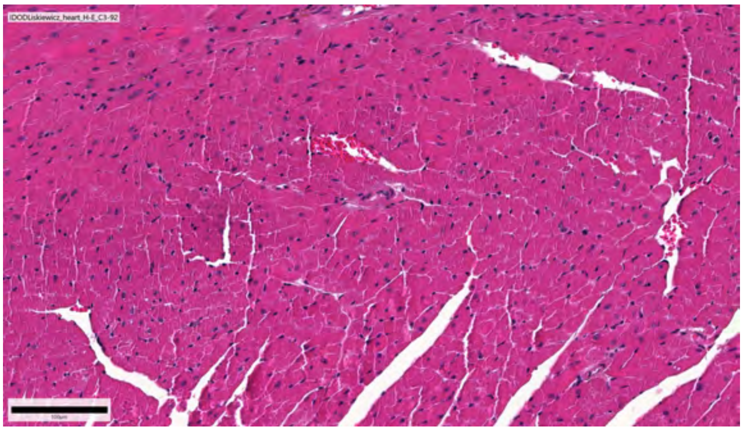

Conjugate #3

Original Pictures for Figure 3l

heart

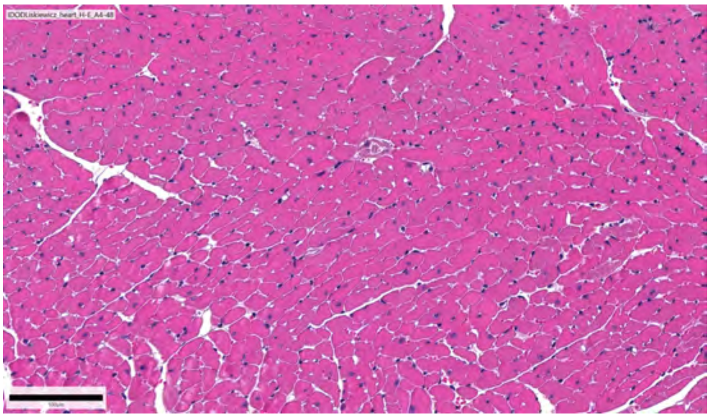

Vhcl #1

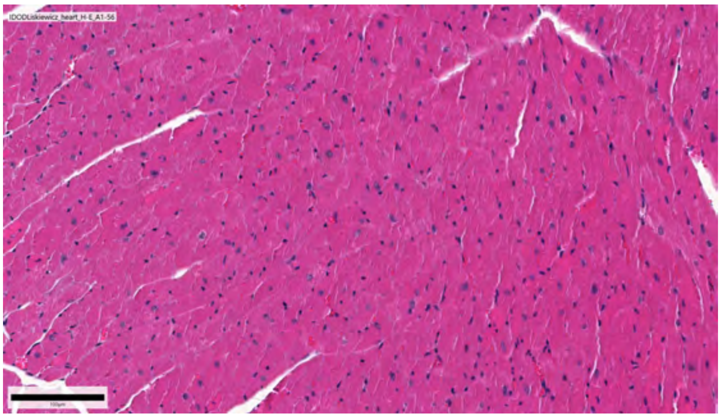

Vhcl #2

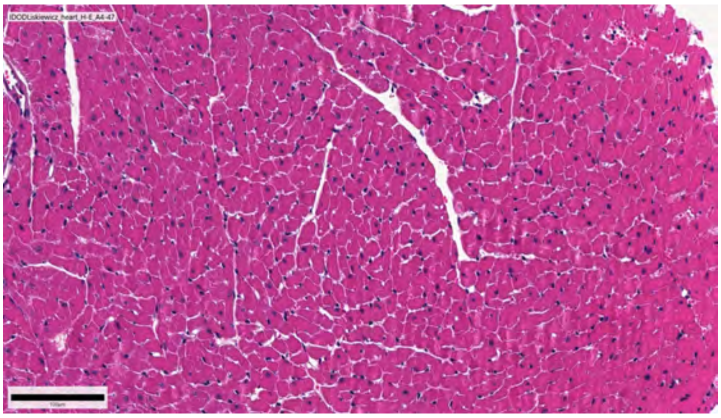

Vhcl #3

Original Pictures for Figure 3l

kidney

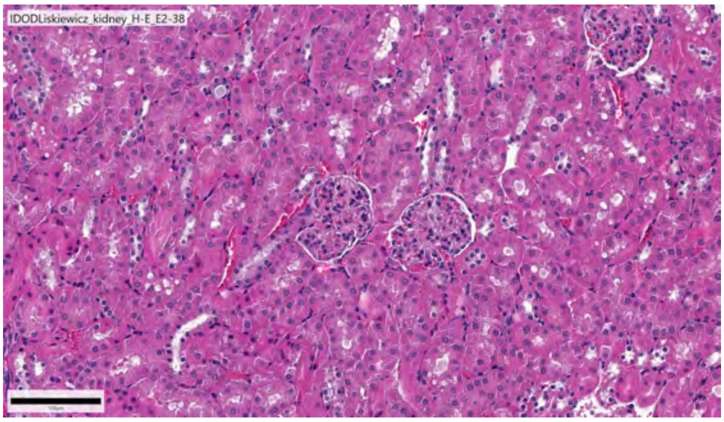

Co-Therapy #1

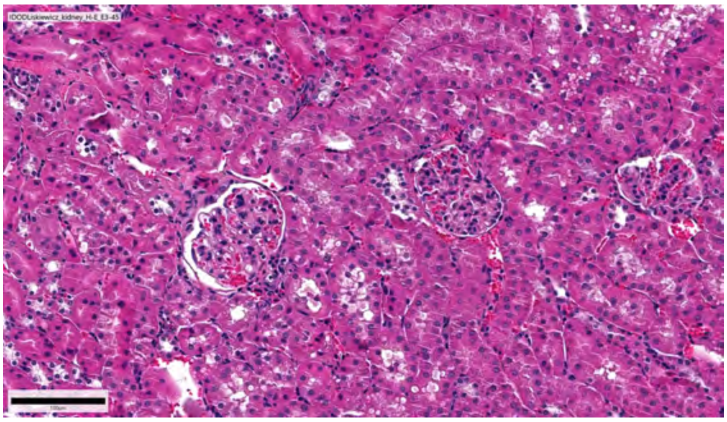

Co-Therapy #2

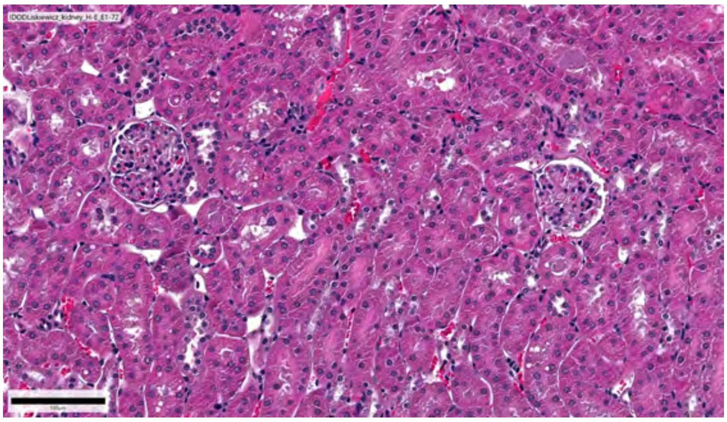

Co-Therapy #3

Original Pictures for Figure 3l

kidney

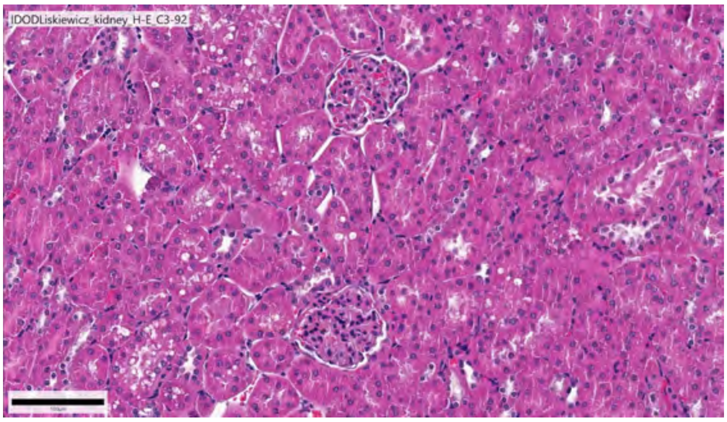

Conjugate #1

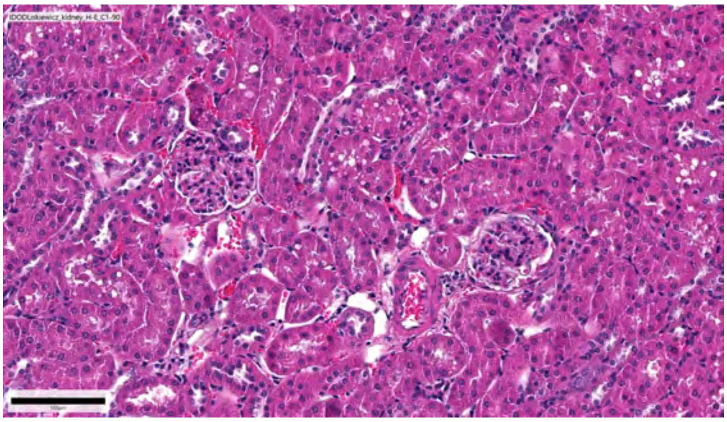

Conjugate #2

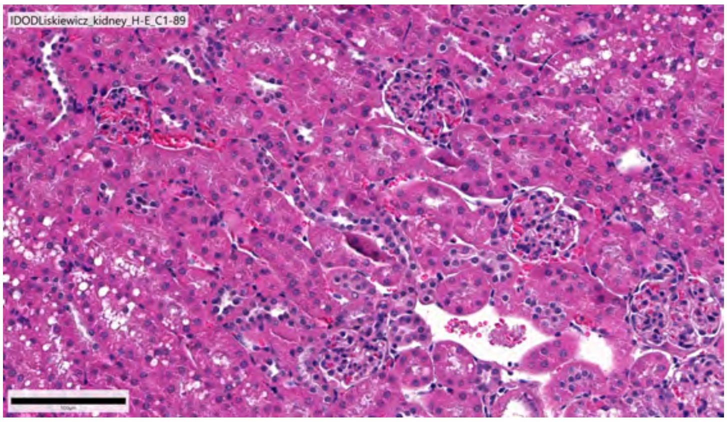

Conjugate #3

Original Pictures for Figure 3l

kidney

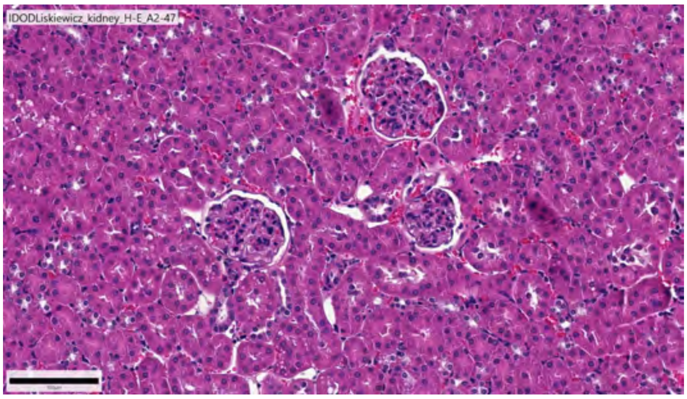

Vhcl #1

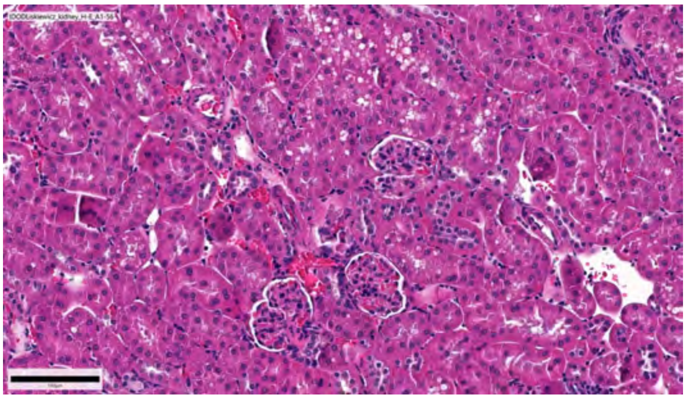

Vhcl #2

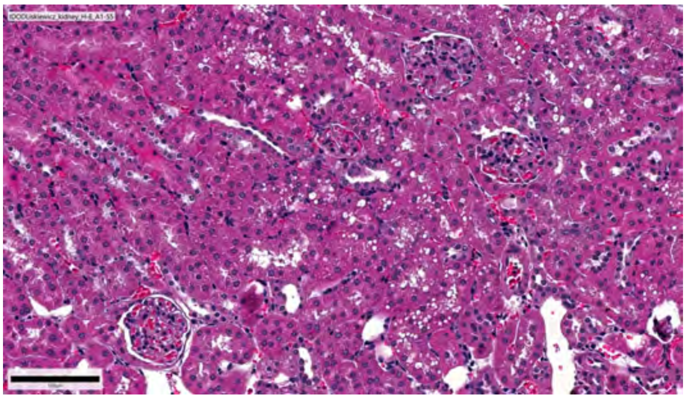

Vhcl #3

Original Pictures for Figure 3l

liver

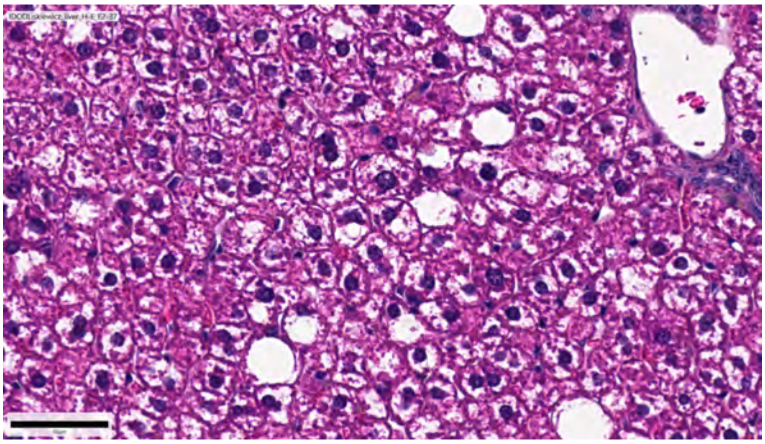

Co-Therapy #1

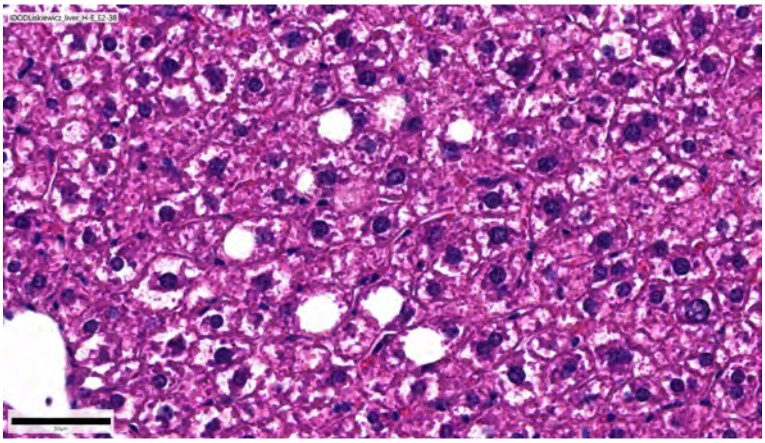

Co-Therapy #2

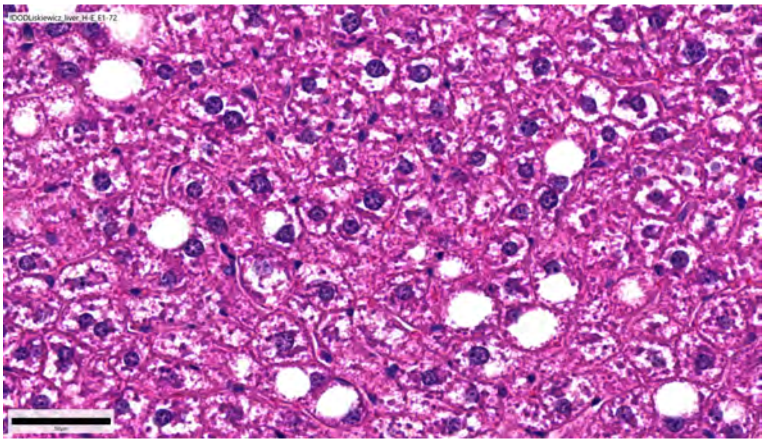

Co-Therapy #3

Original Pictures for Figure 3l

liver

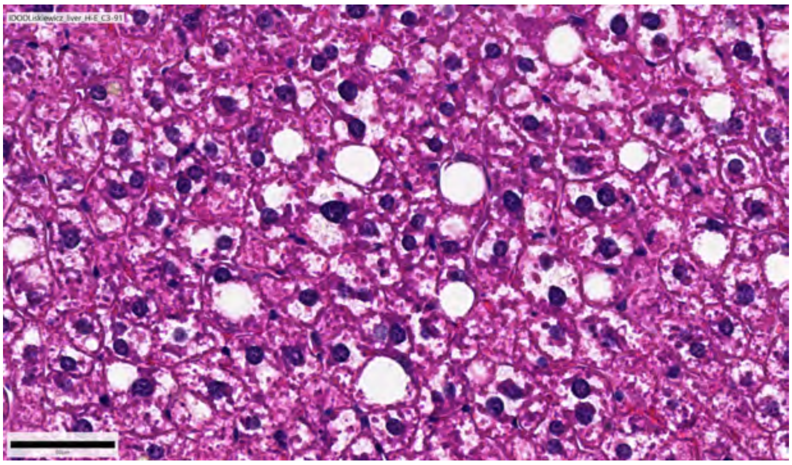

Conjugate #1

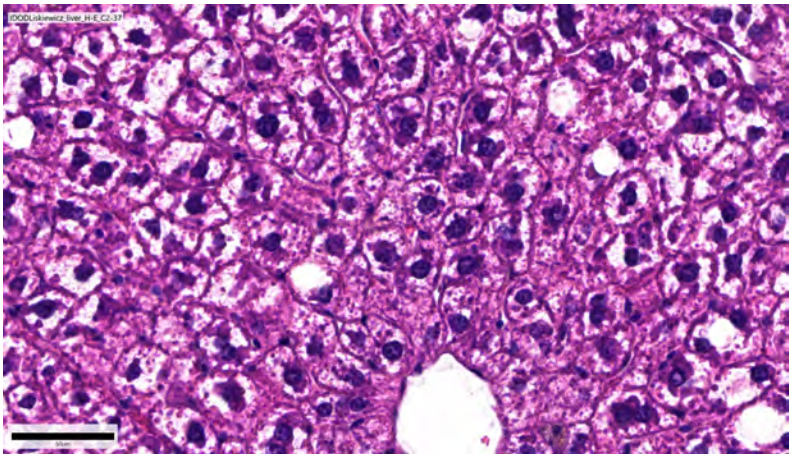

Conjugate #2

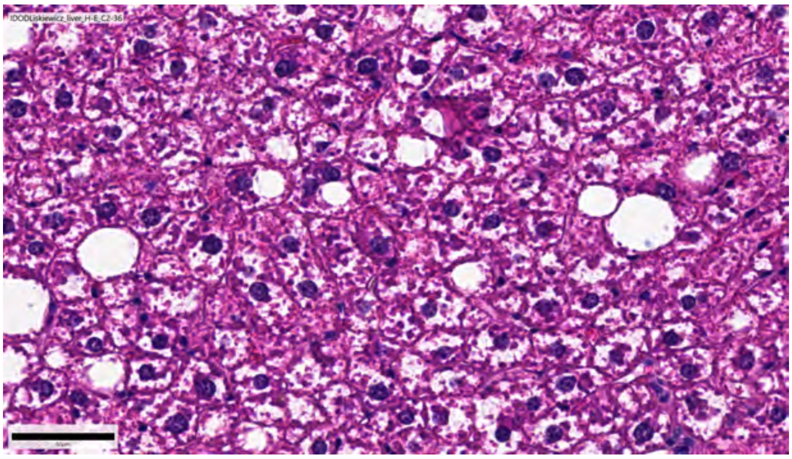

Conjugate #3

Original Pictures for Figure 3l

Liver

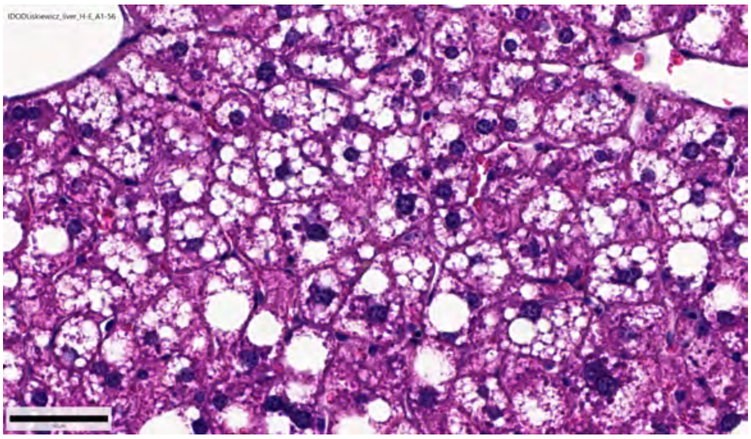

Vhcl #1

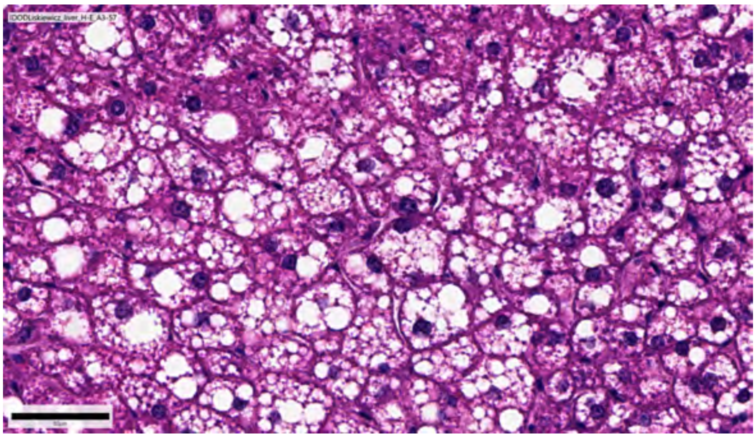

Vhcl #2

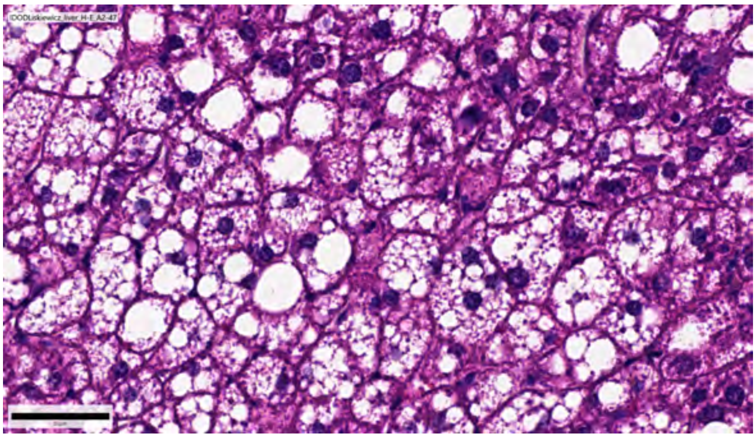

Vhcl #3

Original Pictures for Figure 3l

pancreas

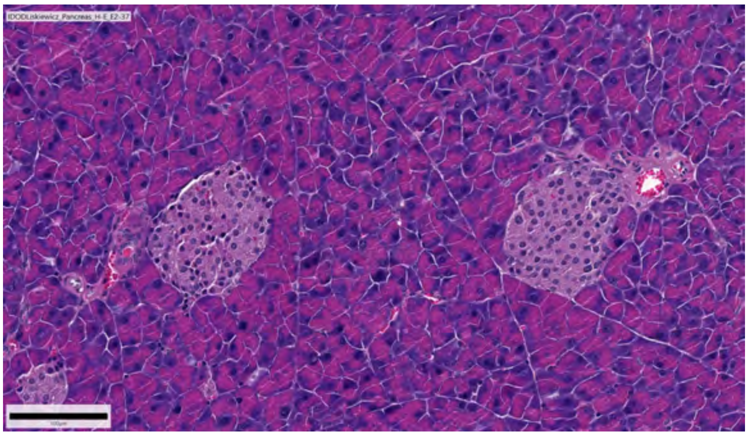

Co-Therapy #1

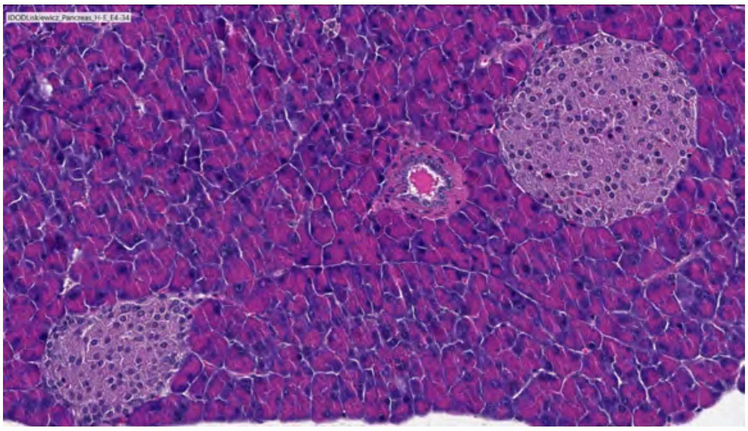

Co-Therapy #2

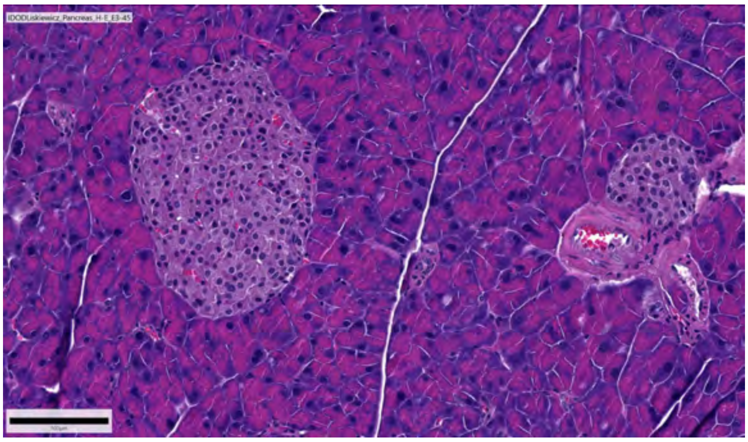

Co-Therapy #3

Original Pictures for Figure 3l

pancreas

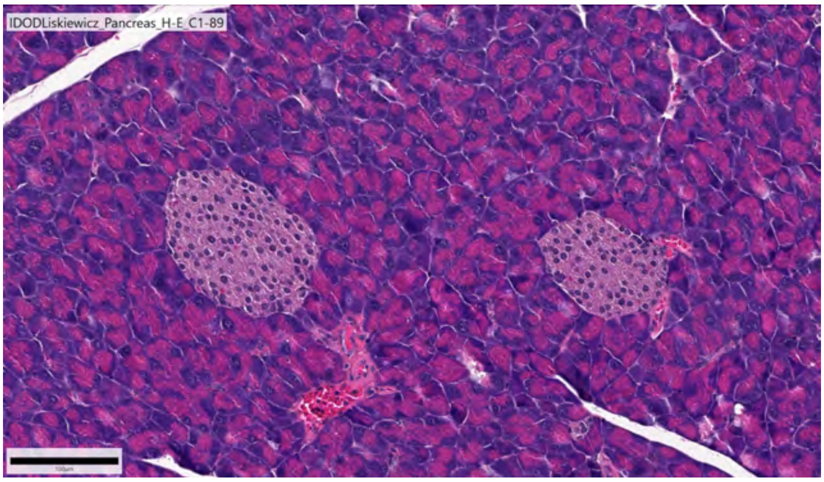

Conjugate #1

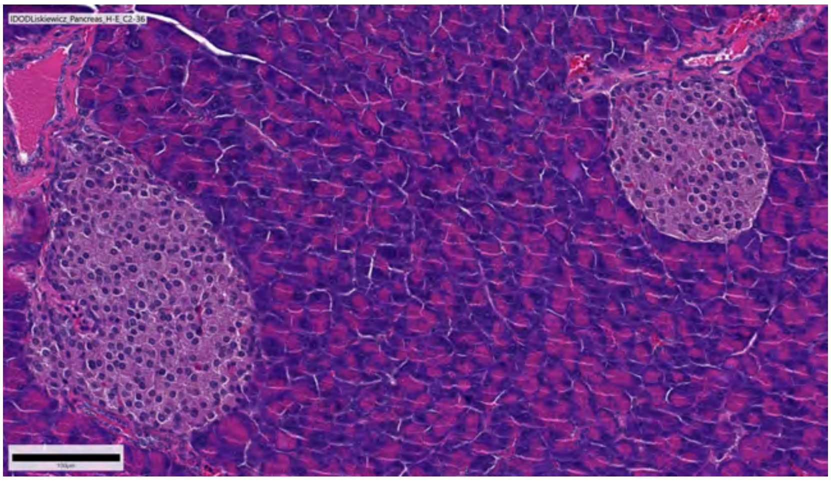

Conjugate #2

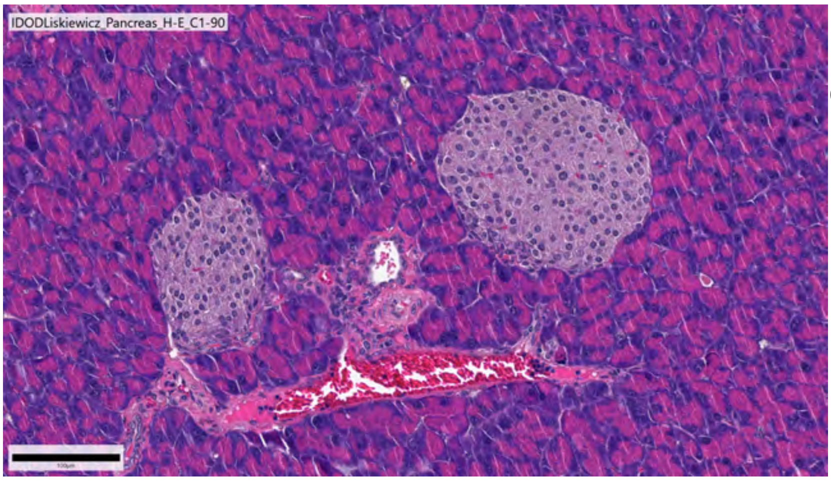

Conjugate #3

Original Pictures for Figure 3l

pancreas

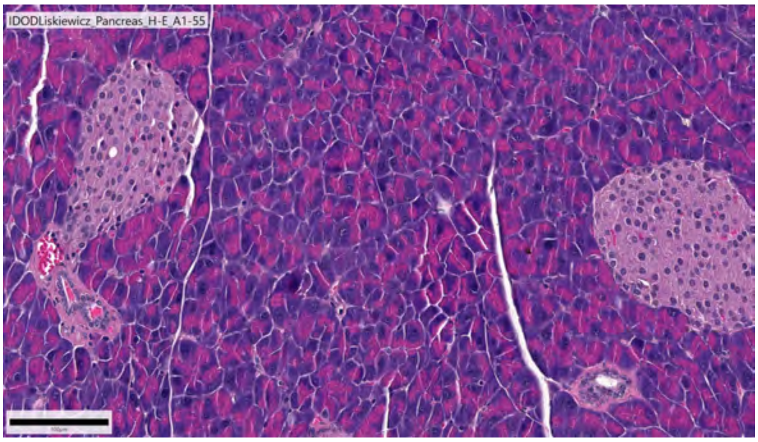

Vhcl #1

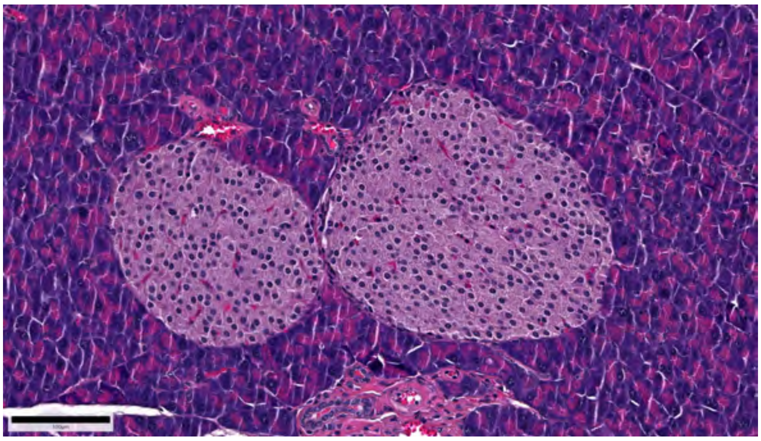

Vhcl #2

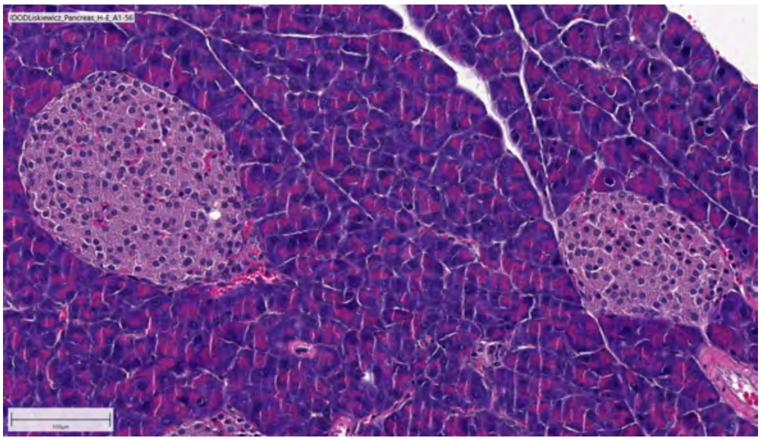

Vhcl #3

Original Pictures for Figure 3l

quadriceps

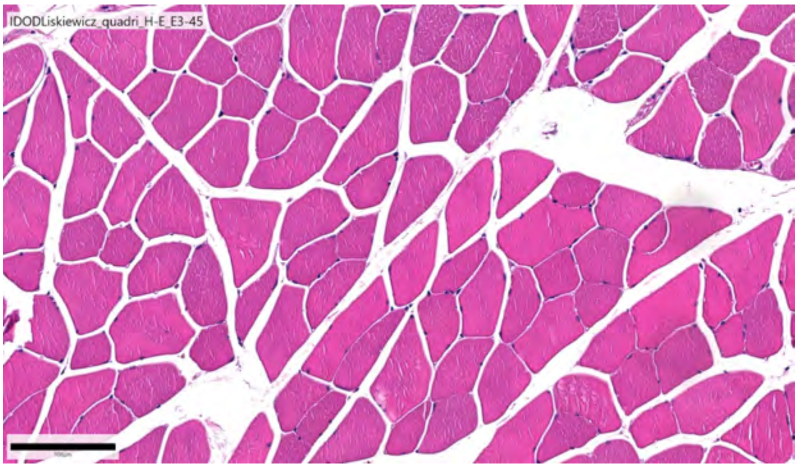

Co-Therapy #1

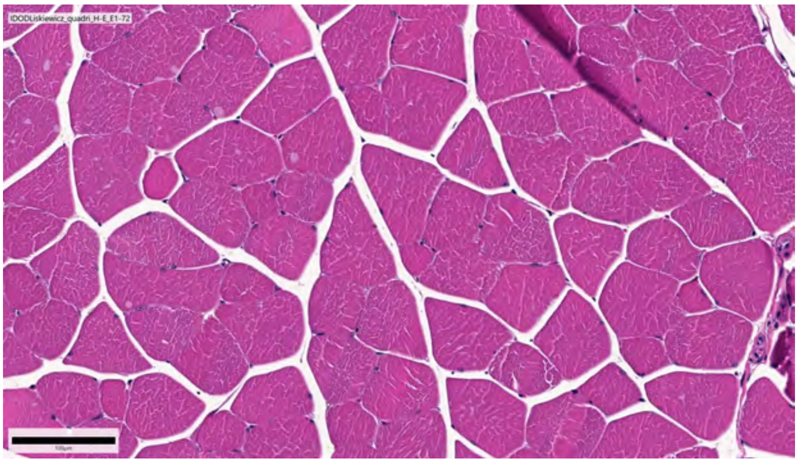

Co-Therapy #2

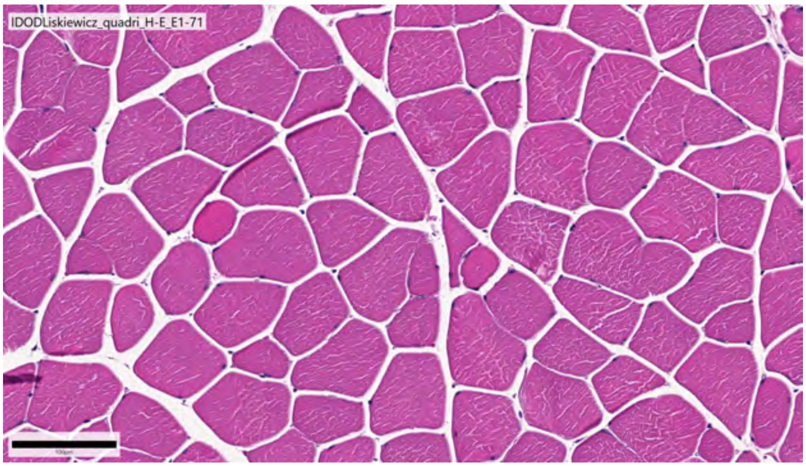

Co-Therapy #3

Original Pictures for Figure 3l

quadriceps

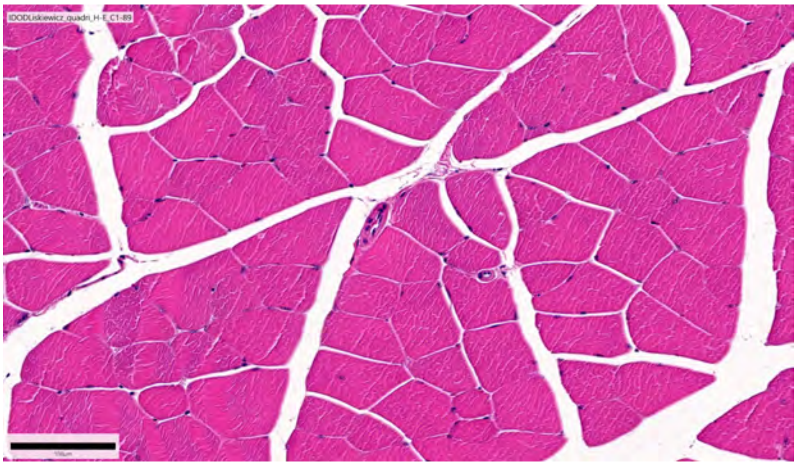

Conjugate #1

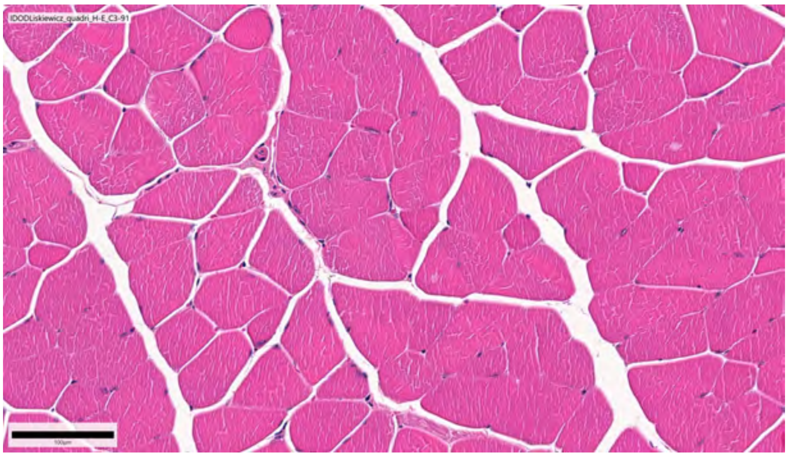

Conjugate #2

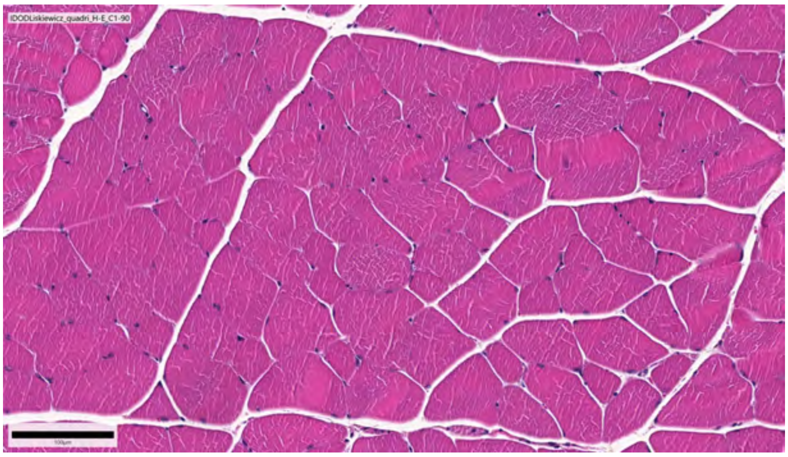

Conjugate #3

Original Pictures for Figure 3l

quadriceps

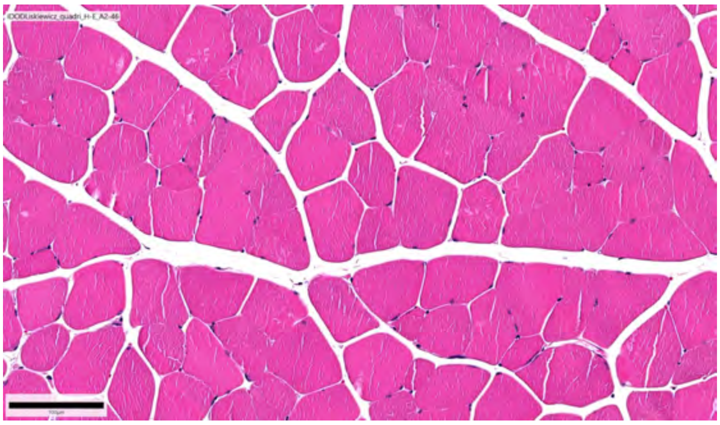

Vhcl #1

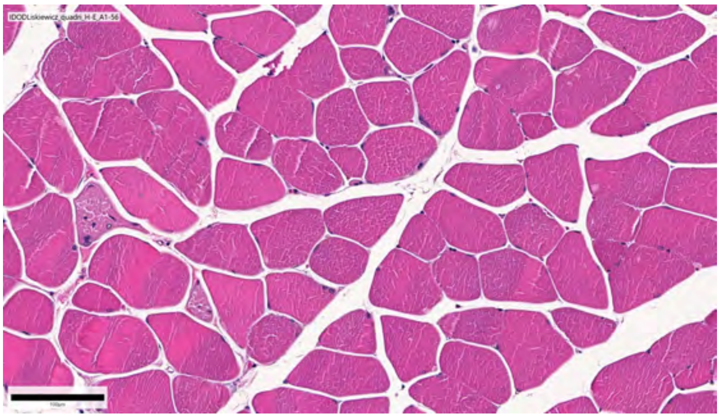

Vhcl #2

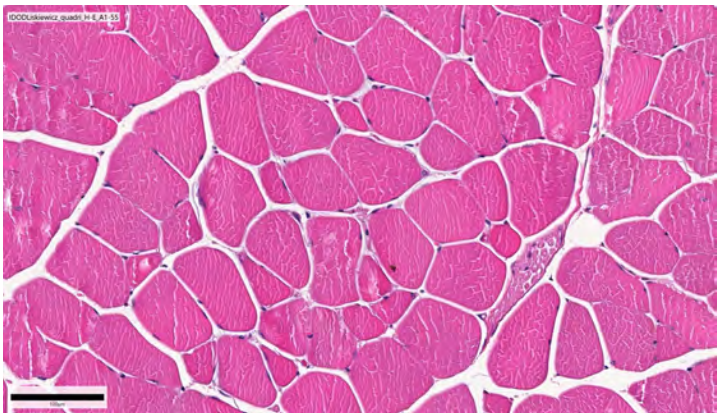

Vhcl #3

Original Pictures for Figure 5a,e,f

GLP-1:GIP #1

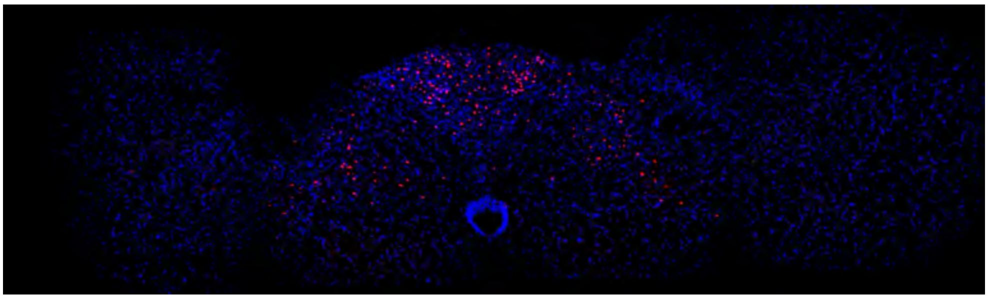

GLP-1:GIP #2

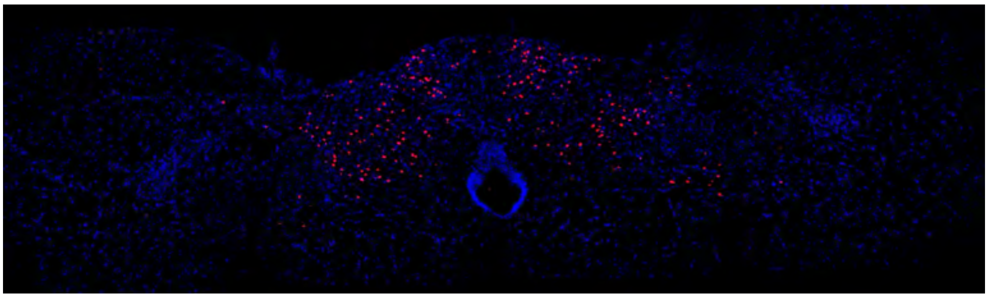

GLP-1:GIP #3

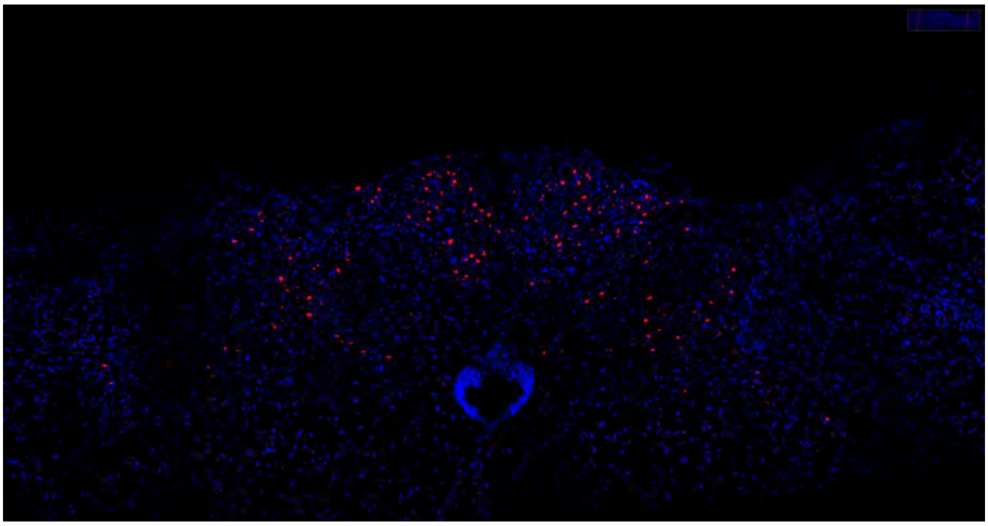

GLP-1:GIP #4

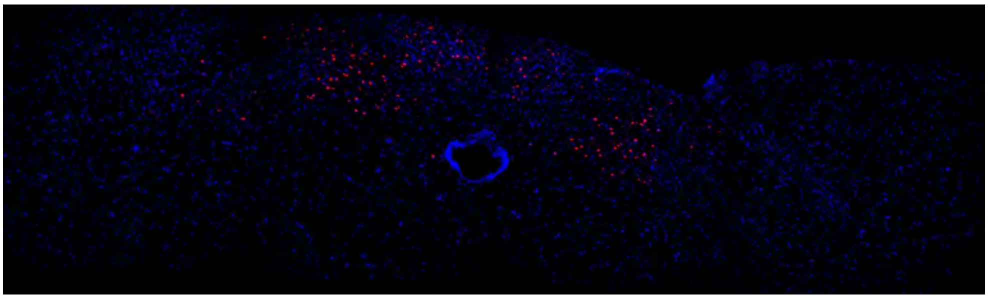

Original Pictures for Figure 5a,e,f

GLP-1:GIP:Lani #1

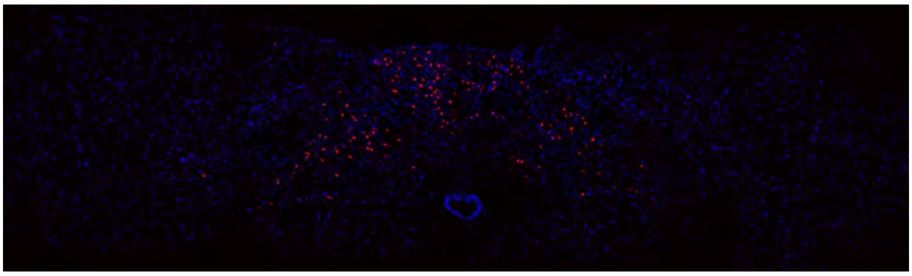

GLP-1:GIP:Lani #2

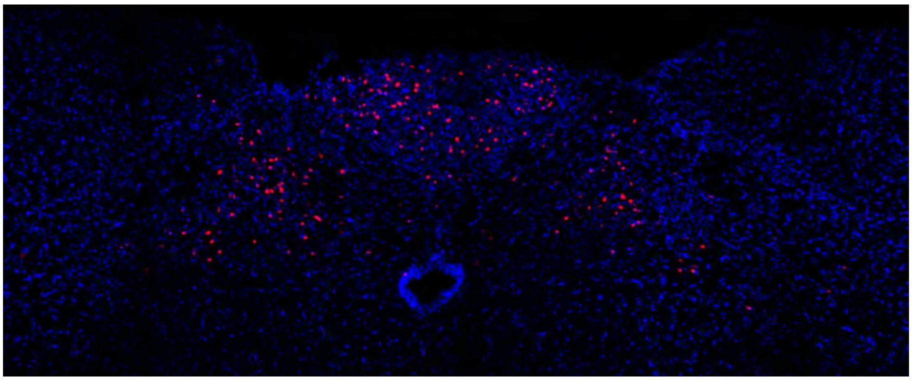

GLP-1:GIP:Lani #3

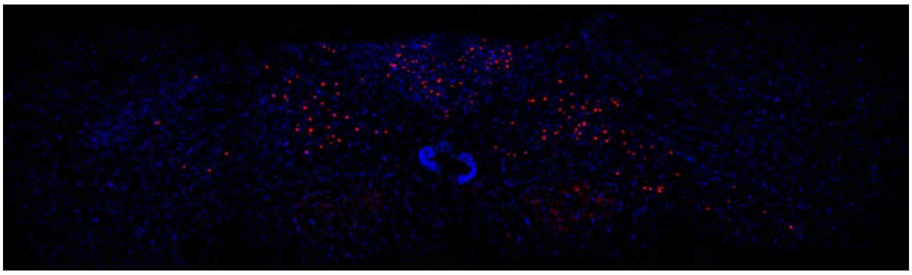

GLP-1:GIP:Lani #4

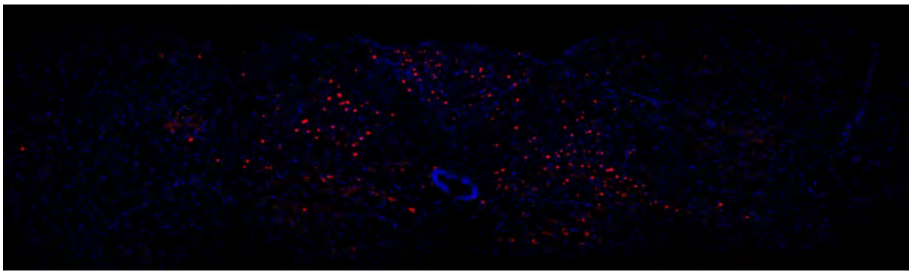

Original Pictures for Figure 5a,e,f

Lani #1

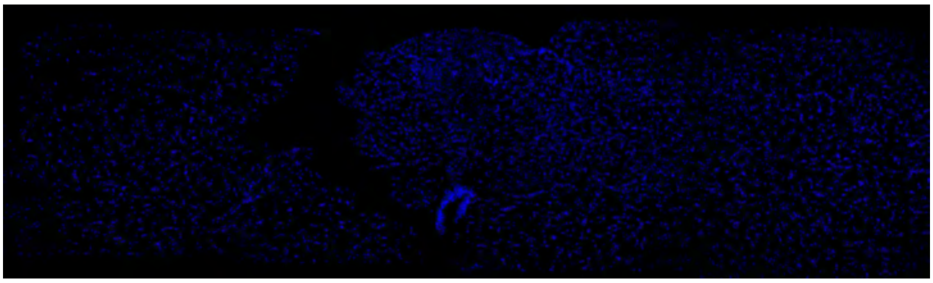

Lani #2

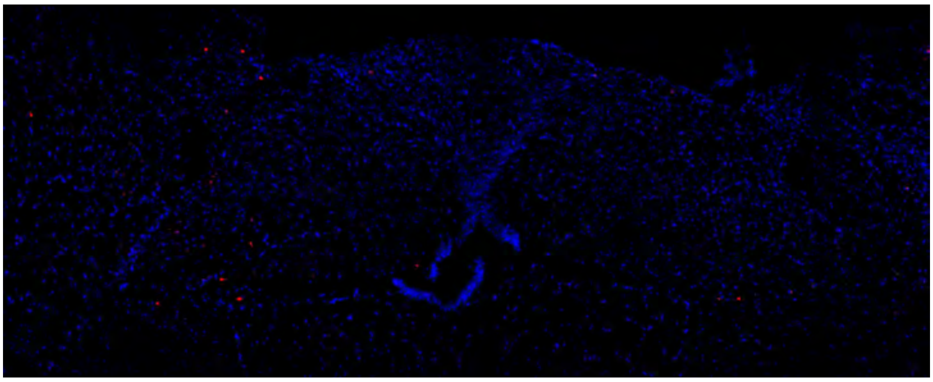

Lani #3

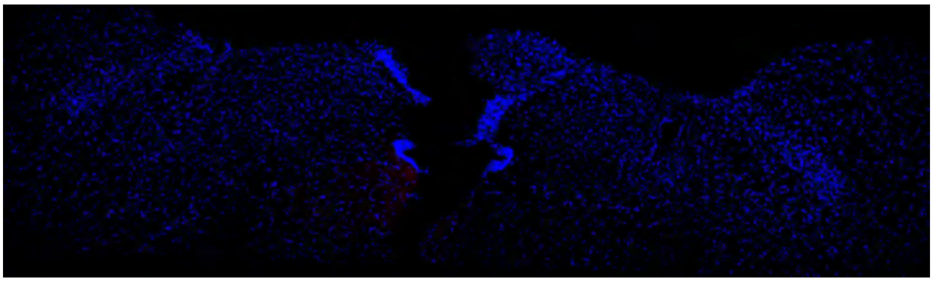

Lani #4

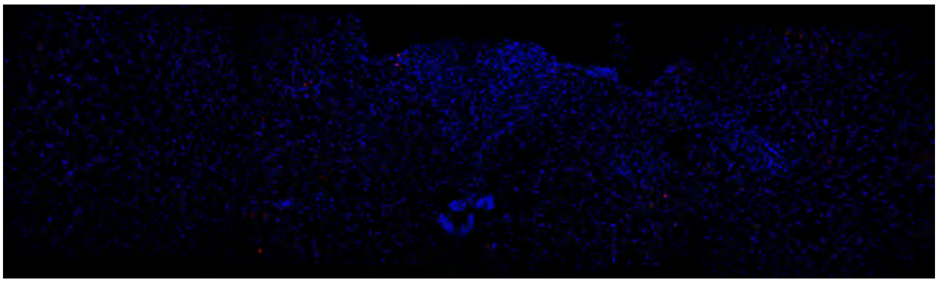

Original Pictures for Figure 5a,e,f

Vhcl #1

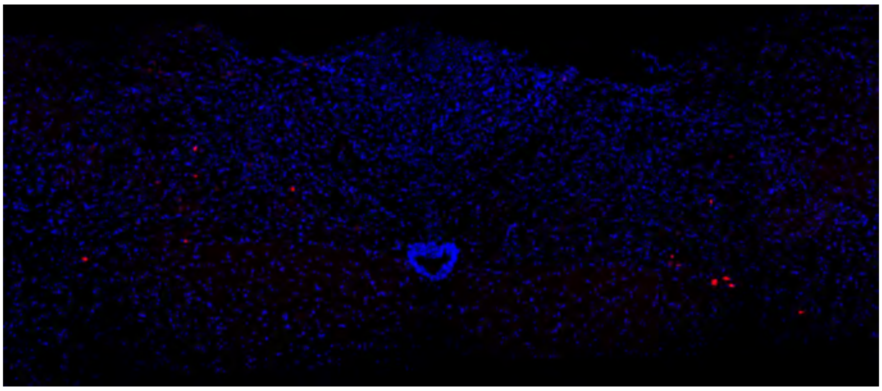

Vhcl #2

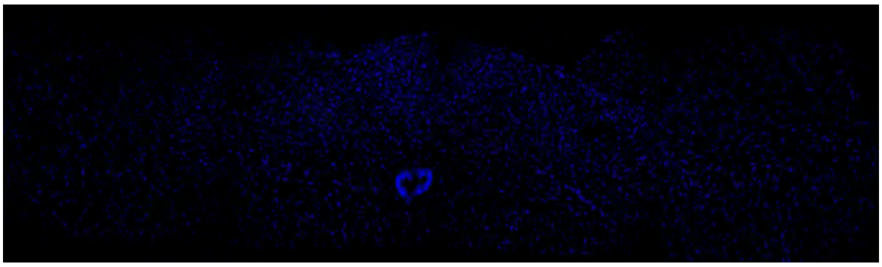

Vhcl #3

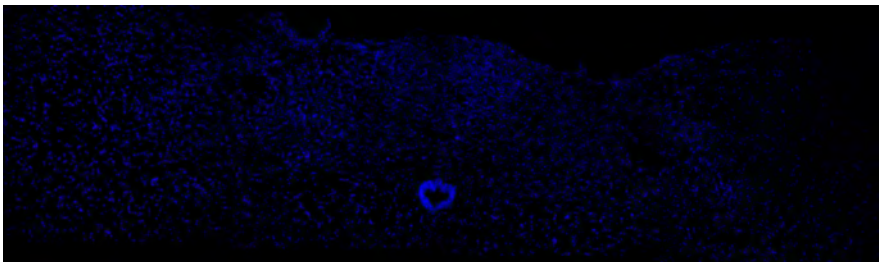

Vhcl #4

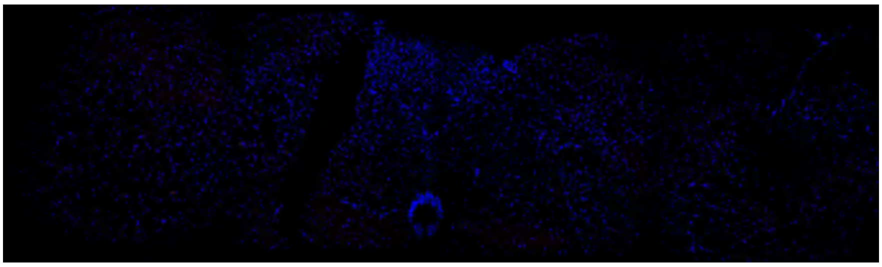

Original Pictures for Figure 5b,c

GLP-1:GIP #1

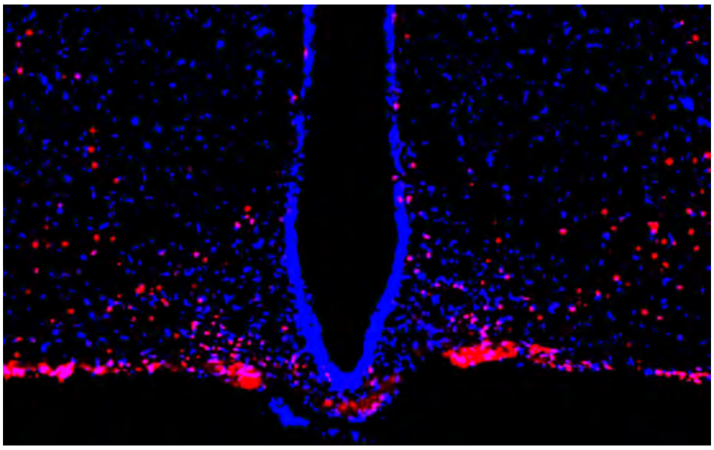

GLP-1:GIP #2

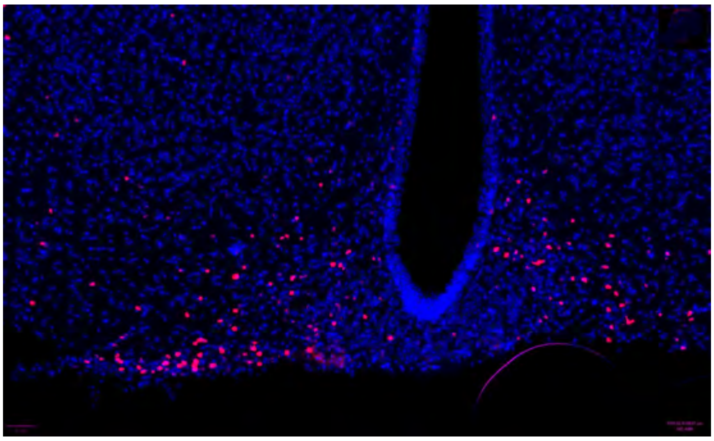

GLP-1:GIP #3

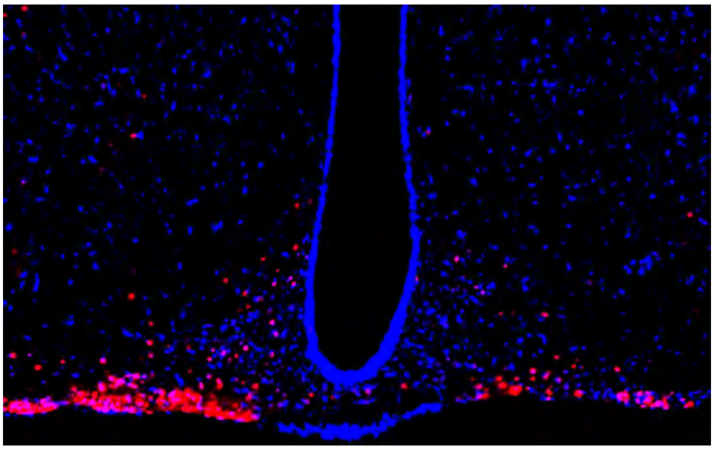

GLP-1:GIP #4

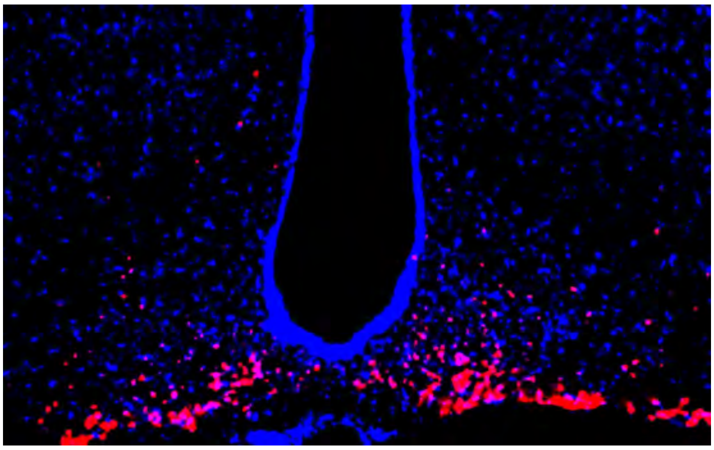

Original Pictures for Figure 5b,c

GLP-1:GIP:Lani #1

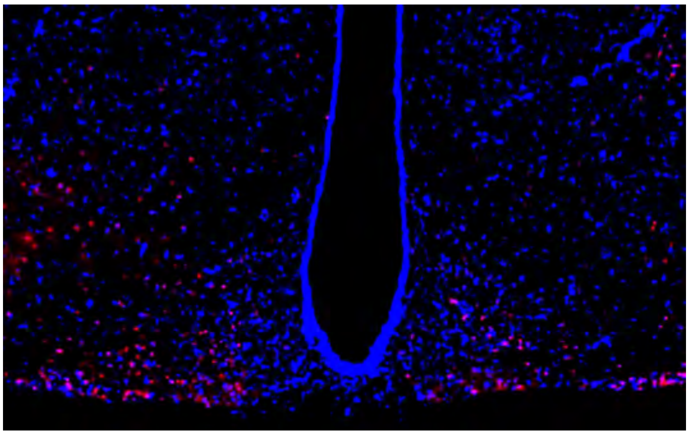

GLP-1:GIP:Lani #2

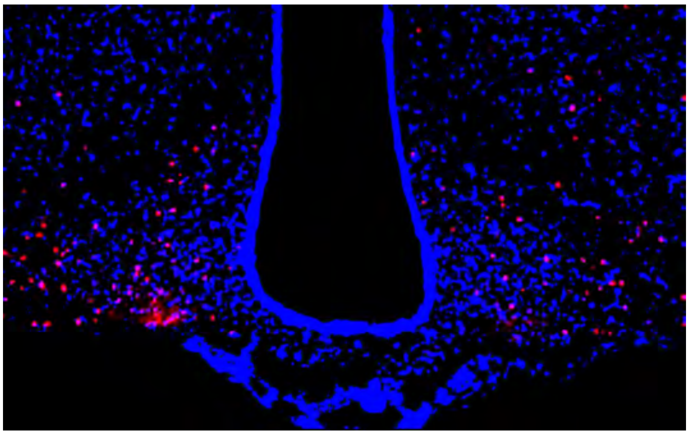

GLP-1:GIP:Lani #3

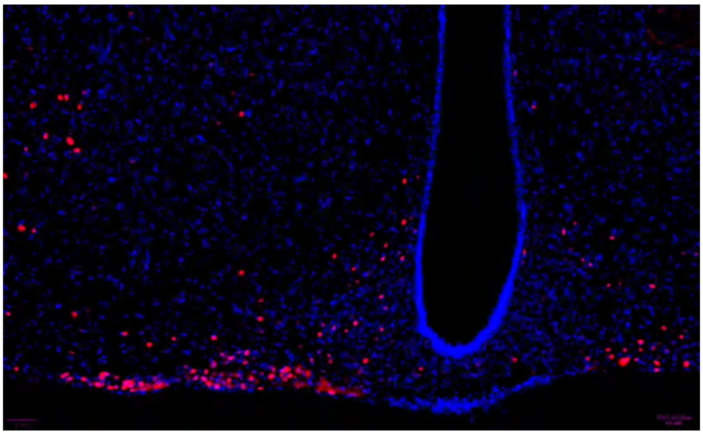

Original Pictures for Figure 5b,c

Lani #1

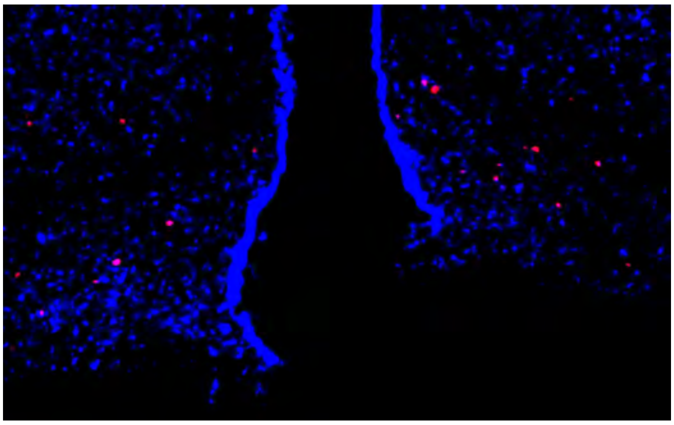

Lani #2

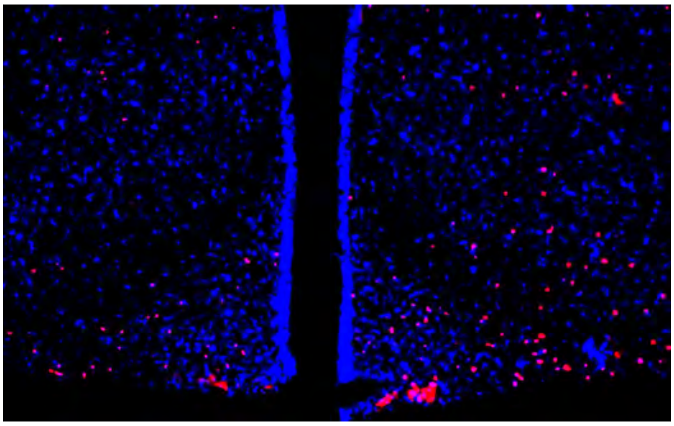

Lani #3

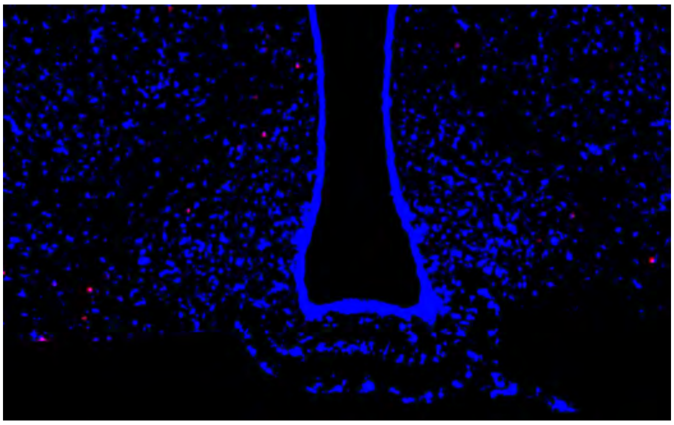

Lani #4

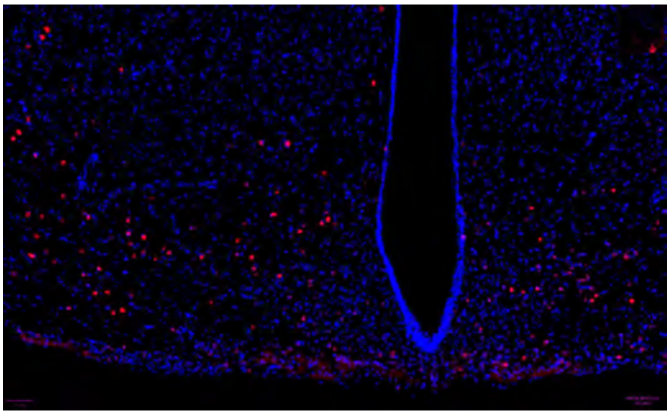

Original Pictures for Figure 5b,c

Vhcl #1

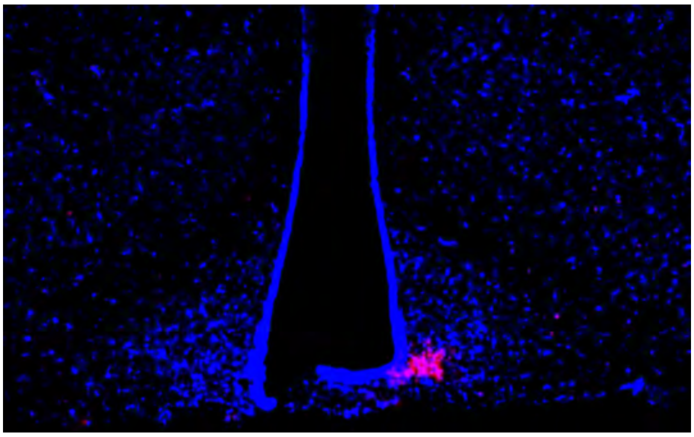

Vhcl #2

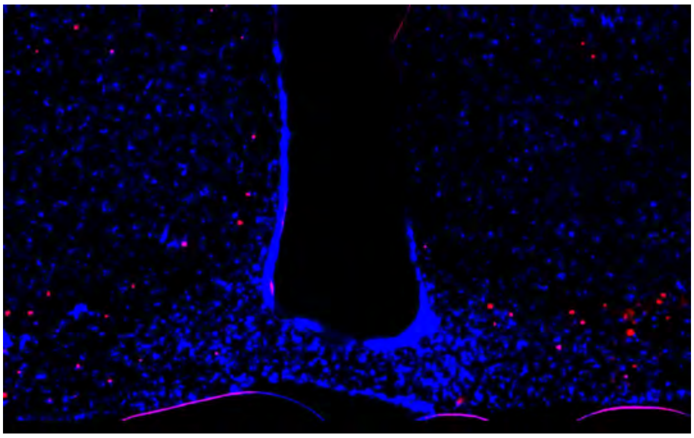

Vhcl #3

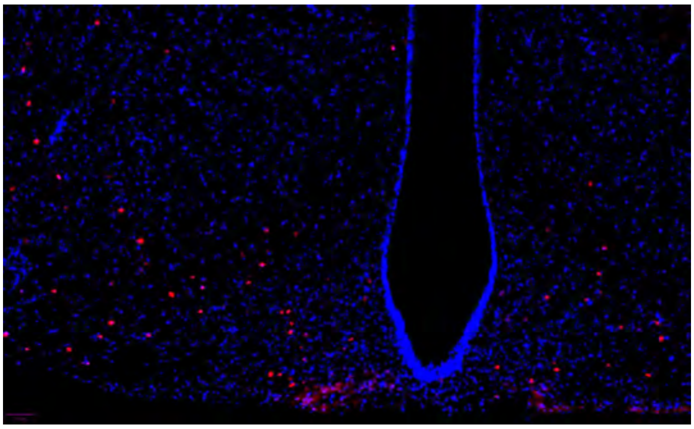

Vhcl #4

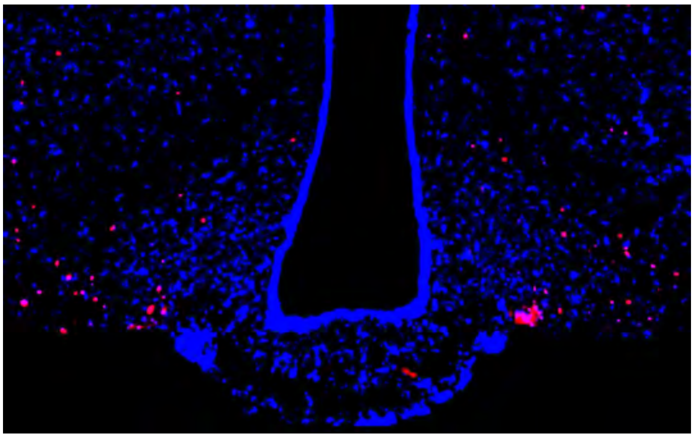

Original Pictures for Figure 5d

GLP-1:GIP #1

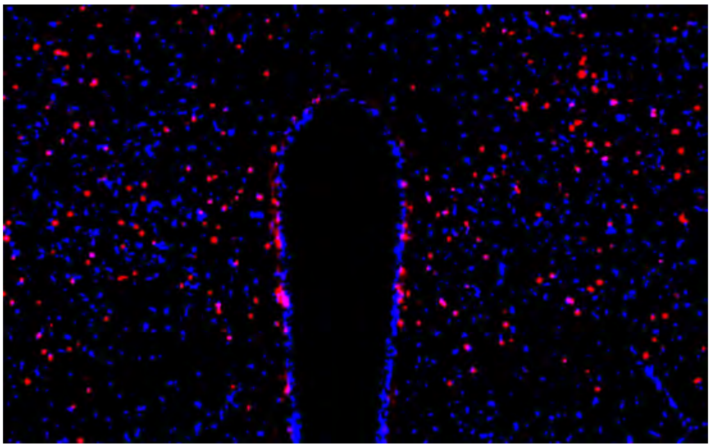

GLP-1:GIP #2

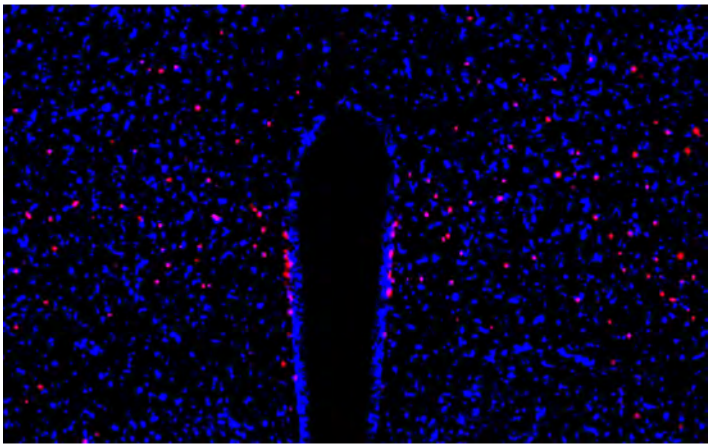

GLP-1:GIP #3

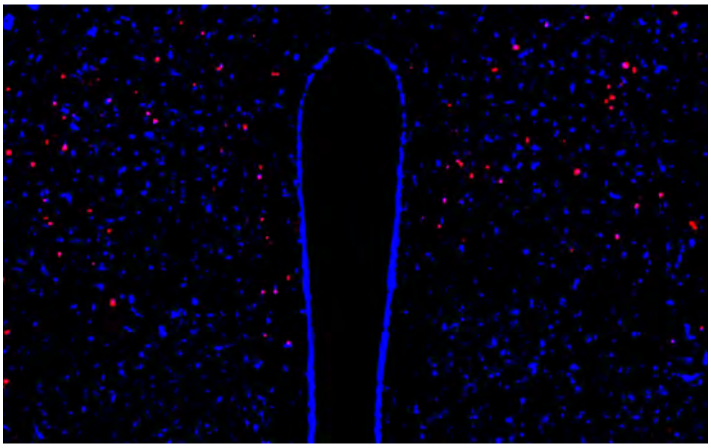

GLP-1:GIP #4

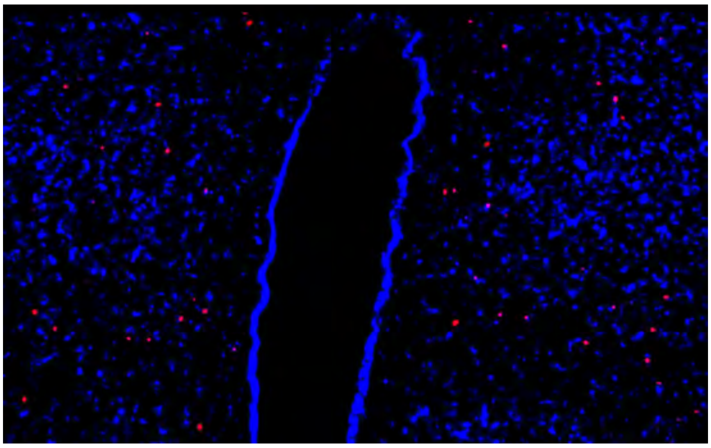

Original Pictures for Figure 5d

GLP-1:GIP:Lani #1

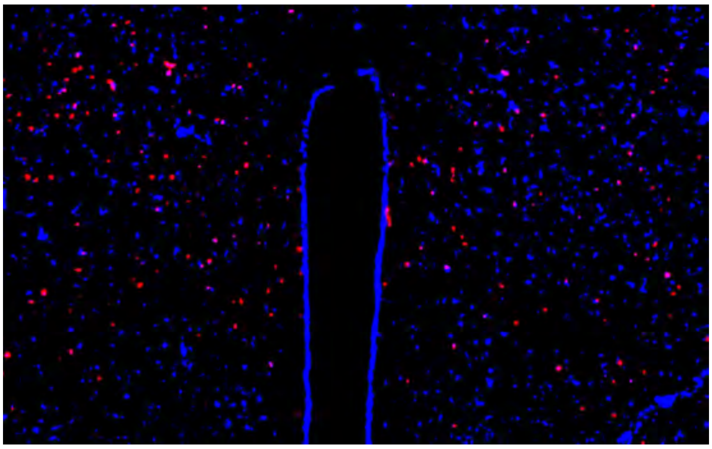

GLP-1:GIP:Lani #2

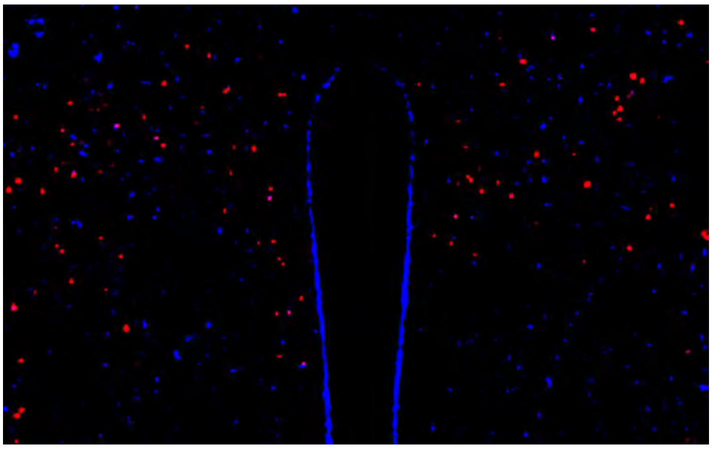

GLP-1:GIP:Lani #3

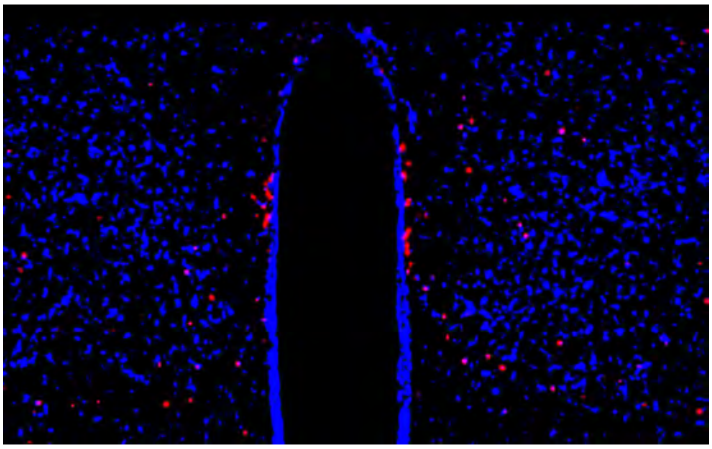

Original Pictures for Figure 5d

Lani #1

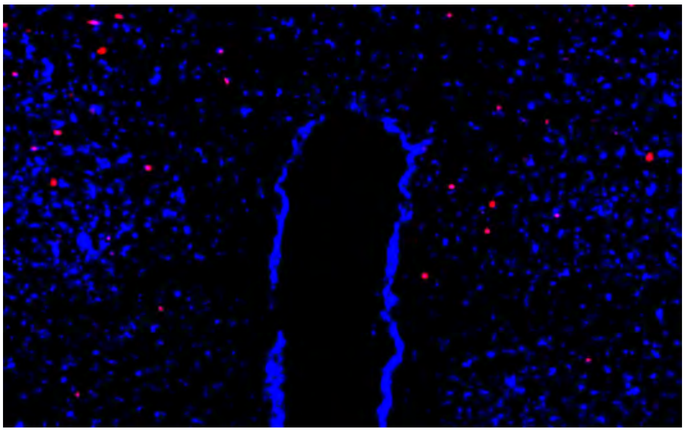

Lani #2

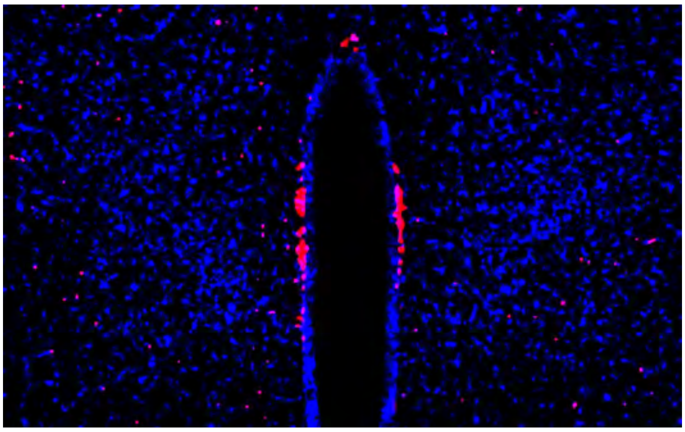

Lani #3

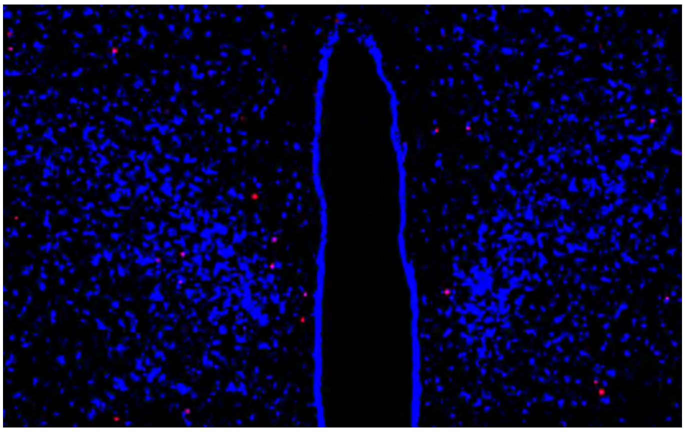

Lani #4

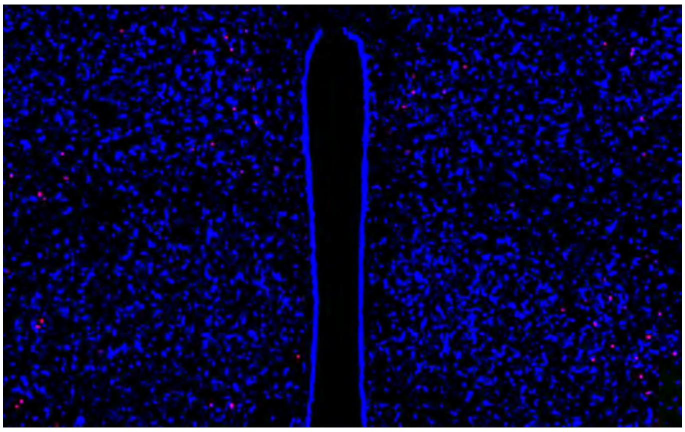

Original Pictures for Figure 5d

Vhcl #1

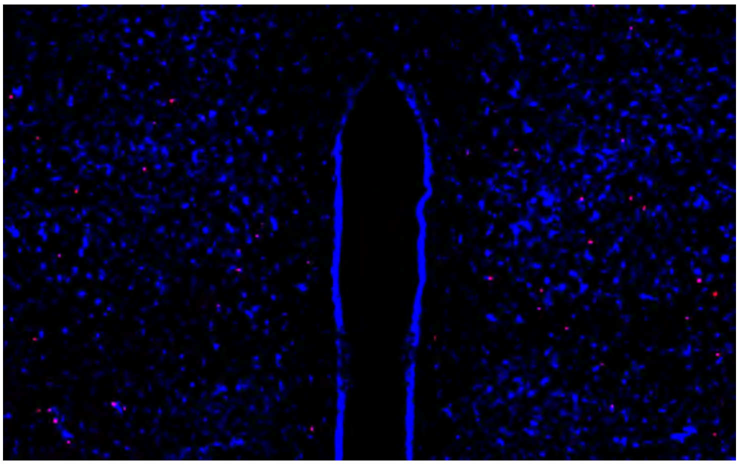

Vhcl #2

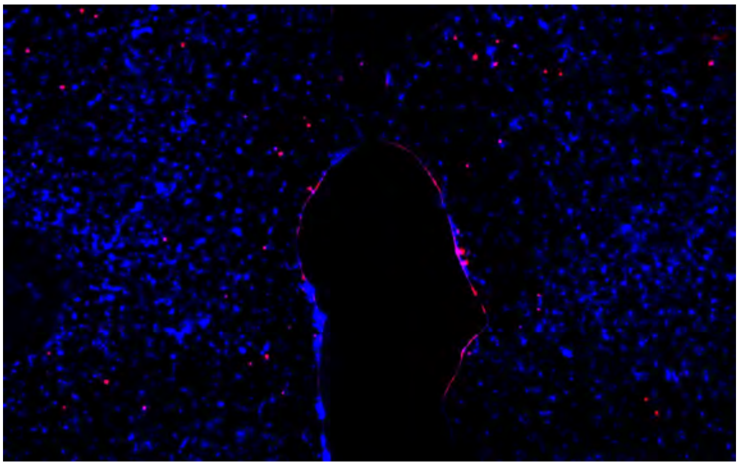

Vhcl #3

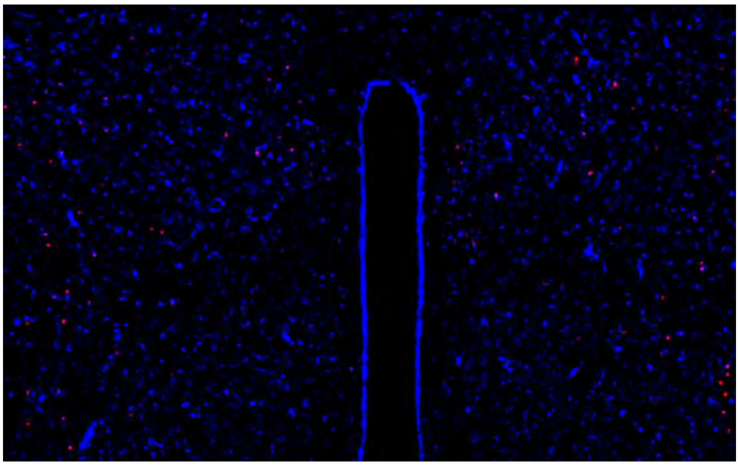

Vhcl #4

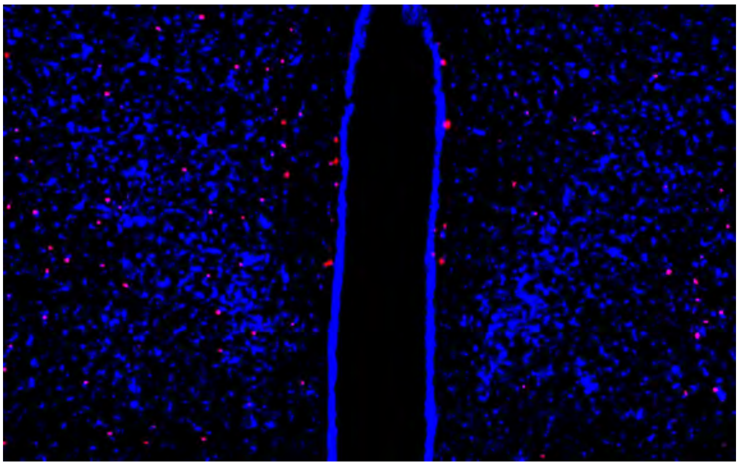

Supplement: Supplementary file 3 — Original histological or microscopic pictures for Figs. 2p, 3l and 5a–f. [file 41586_2026_10427_MOESM3_ESM.pdf]
